# Ultra‐High Performance Amorphous Ga_2_O_3_ Photodetector Arrays for Solar‐Blind Imaging

**DOI:** 10.1002/advs.202101106

**Published:** 2021-08-13

**Authors:** Yuan Qin, Li‐Heng Li, Zhaoan Yu, Feihong Wu, Danian Dong, Wei Guo, Zhongfang Zhang, Jun‐Hui Yuan, Kan‐Hao Xue, Xiangshui Miao, Shibing Long

**Affiliations:** ^1^ Key Laboratory of Microelectronics Devices & Integration Technology Institute of Microelectronics of Chinese Academy of Sciences Beijing 100029 China; ^2^ School of Microelectronics University of Science and Technology of China Hefei Anhui 230026 China; ^3^ Wuhan National Laboratory for Optoelectronics School of Optical and Electronic Information Huazhong University of Science and Technology Wuhan 430074 China

**Keywords:** Ga_2_O_3_, high detectivity, image sensors, photodetector arrays, solar‐blind imaging, uniformity

## Abstract

The growing demand for scalable solar‐blind image sensors with remarkable photosensitive properties has stimulated the research on more advanced solar‐blind photodetector (SBPD) arrays. In this work, the authors demonstrate ultrahigh‐performance metal‐semiconductor‐metal (MSM) SBPDs based on amorphous (*a*‐) Ga_2_O_3_ via a post‐annealing process. The post‐annealed MSM *a*‐Ga_2_O_3_ SBPDs exhibit superhigh sensitivity of 733 A/W and high response speed of 18 ms, giving a high gain‐bandwidth product over 10^4^ at 5 V. The SBPDs also show ultrahigh photo‐to‐dark current ratio of 3.9 × 10^7^. Additionally, the PDs demonstrate super‐high specific detectivity of 3.9 × 10^16^ Jones owing to the extremely low noise down to 3.5 fW Hz^−1/2^, suggesting high signal‐to‐noise ratio. Underlying mechanism for such superior photoelectric properties is revealed by Kelvin probe force microscopy and first principles calculation. Furthermore, for the first time, a large‐scale, high‐uniformity 32 × 32 image sensor array based on the post‐annealed *a*‐Ga_2_O_3_ SBPDs is fabricated. Clear image of target object with high contrast can be obtained thanks to the high sensitivity and uniformity of the array. These results demonstrate the feasibility and practicality of the Ga_2_O_3_ PDs for applications in solar‐blind imaging, environmental monitoring, artificial intelligence and machine vision.

## Graphical abstract

Ultraviolet imaging technology is widely used in meteorology, medical science, and military science. For the first time, a high‐uniformity 32 × 32 solar‐blind image sensor array with outstanding imaging capability is demonstrated based on high‐performance Ga_2_O_3_ photodetectors. Schottky barrier lowering effect is experimentally revealed to attribute to the internal gain mechanism.

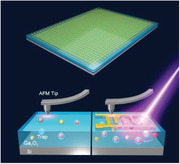

## 1 Introduction

Owing to the extensive applications in modern industry and agriculture, photodetectors (PDs),^[^ ^1^, ^2^, ^3^ ^]^ light‐emitting diodes,^[^ ^4^ ^]^ and other optoelectronic devices^[^ ^5^ ^]^ have attracted intensive attention in recent years.^[^ ^6^, ^7^, ^8^, ^9^ ^]^ With the development of emerging wide band gap semiconductors, such as BN, Al*_x_* Ga_1−_ *_x_* N, Ga_2_O_3_ and SiC, vast research interests have been focused on solar‐blind PDs (SBPDs) for their excellent radiation hardness, high thermal and chemical stabilities, as well as efficient absorption in solar‐blind region.^[^ ^6^, ^10^, ^11^, ^12^ ^]^ Among these materials, Ga_2_O_3_ has an absorption cut‐off wavelength below 280 nm, covering almost the entire range of solar‐blind region without the need of alloying. These special properties render Ga_2_O_3_ a promising candidate for SBPDs.

Recently, significant breakthroughs based on different types of Ga_2_O_3_ PDs have been reported.^[^ ^6^, ^13^, ^14^, ^15^, ^16^, ^17^, ^18^, ^19^, ^20^ ^]^ P‐type semiconductors, such as GaN and NiO,^[^ ^15^, ^21^, ^22^ ^]^ were employed to make p–n heterojunction with Ga_2_O_3_ towards photodetection usages. Tang et al. constructed self‐driven UV PDs with outstanding photosensitive performance by optimizing the growth condition of Sn‐doped n‐type Ga_2_O_3_ on p‐type GaN film.^[^ ^15^ ^]^ Three‐terminal phototransistor is another alternative to construct highly sensitive PDs with high intrinsic gain. As demonstrated in several previous works,^[^ ^18^, ^23^, ^24^ ^]^ it is very facile to achieve high responsivity (*R*) and rejection ratio in Ga_2_O_3_ phototransistors. Additionally, the response speed can be modulated by the gate terminal. Schottky junction is also commonly used in Ga_2_O_3_ PDs. Ahn et al. utilized MgO to fabricate *β*‐Ga_2_O_3_/MgO heterostructure‐based phototransistor, achieving high performances such as an ultrahigh responsivity of 2.4 × 10^7^ A W^−1^ and a specific detectivity of 1.7 × 10^15^ Jones.^[^ ^25^ ^]^ Yu et al. constructed a solar‐blind phototransistor based on *β*‐Ga_2_O_3_ micro flake. The ultra‐high detectivity of 1.19 × 10^18^ Jones makes it extremely suitable for weak light detection.^[^ ^26^ ^]^ Li et al. adopted high‐k dielectric–hafnium oxide (HfO_2_) to construct a quite outstanding metal‐oxide‐semiconductor field‐effect phototransistor. The fabricated device achieved a record‐high detectivity of 1.1 × 10^19^ Jones, exceptionally high responsivity of 1.4 × 10^7^ A W^−1^, as well as a short decay time of 16 ms.^[^ ^27^ ^]^ Recently, Xu et al.^[^ ^28^ ^]^ reported a high‐speed Ga_2_O_3_ Schottky photodiode with large light absorption area. The high speed and high detectivity could enable real‐time imaging if dark current is further optimized. In particular, the metal–semiconductor–metal (MSM) PDs can be fabricated with the simplest processes.^[^ ^29^ ^]^ Aside from its ordinary structure, MSM PDs do not require the formation of Ohmic contact, which in general involves complicated doping process. Moreover, device isolation is also not required for MSM PDs. Even with such simplified fabrication processes, MSM PDs can well maintain their high performances such as low noise and high gain‐bandwidth product. However, few reports are available for large‐area Ga_2_O_3_ PD array for solar‐blind imaging.

The key challenges for large‐area Ga_2_O_3_ PD image sensor array lie in the difficulty of growing large size Ga_2_O_3_ films as well as maintaining their high uniformity in integration. Various methods have been attempted to grow Ga_2_O_3_ films for high‐performance PDs, such as molecular beam epitaxy,^[^ ^30^, ^31^ ^]^ metal‐organic chemical vapor deposition,^[^ ^32^, ^33^, ^34^ ^]^ magnetron sputtering^[^ ^6^, ^13^, ^35^ ^]^ and pulsed laser deposition.^[^ ^36^ ^]^ Among them, magnetron sputtering is particularly suitable for cost‐effective and large scale Ga_2_O_3_ film deposition with smooth surface, based on which high performance Ga_2_O_3_ PDs have been reported. Arora et al.^[^ ^13^ ^]^ deposited *β*‐Ga_2_O_3_ film on Si/SiO_2_ substrate by magnetron sputtering. Through adjusting the growth condition, their *β*‐Ga_2_O_3_ MSM PDs achieved a sensitivity of 96.13 A W^−1^ and low noise of 1.43 pA dark current. Efforts have also been made to realize Ga_2_O_3_ PD arrays. Our group also demonstrated a phototransistor made of amorphous (*a*‐) Ga_2_O_3_ film grown by magnetron sputtering. The feasibility of a single *a*‐Ga_2_O_3_ thin‐film phototransistor as a pixel was successfully verified for imaging application.^[^ ^6^ ^]^ However, in practical applications some drawbacks still persist, such as the low scanning speed and poor spatial recognition of the single pixel imaging. Anamika et al.^[^ ^37^ ^]^ reported a linear MSM PD array based on bulk *β*‐Ga_2_O_3_. The PDs showed satisfactory UV photodetection performance with a peak responsivity and dark current of 4 A W^−1^ and 0.23 nA, respectively. However, the uniformity of device parameters of the linear array is not satisfactory. Peng et al.^[^ ^38^ ^]^ illustrated MSM *β*‐Ga_2_O_3_ PDs through magnetron sputtering and a small 4 × 4 array was fabricated. Unfortunately, further imaging capabilities were not tested or verified. Lu et al.^[^ ^39^ ^]^ also demonstrated a 4 × 4 MSM Ga_2_O_3_ PD array. Due to the high performance and uniformity of the PDs, clear image of target object was obtained.Nevertheless, the wiring method needs to be improved to promote integration of PDs. Luo et al.^[^ ^40^ ^]^ utilized a thermal‐assisted conversion process of metal Ga to *β*‐Ga_2_O_3_ for *β*‐Ga_2_O_3_ film preparation, and fabricated a 8 × 8 photodetector focal plane array. The cells in the array exhibited small fluctuation in dark and photocurrent. Due to the high uniformity of the deposited *β*‐Ga_2_O_3_ film and superior photodetection performance of individual PDs, excellent UV imaging ability was demonstrated. Recently, Lu et al. employed the novel origami method together with the magnetron sputtering process to successfully realize *a*‐Ga_2_O_3_ 3D photosensing array,^[^ ^41^ ^]^ which is quite impressing and represents a significant progress for light trajectory detection and imaging application. However, this method is not very facile for large‐scale integration. Therefore, further works on scalable high‐uniformity and high‐resolution Ga_2_O_3_ image sensor array is urgently needed for the vast applications in large‐area optoelectronics.

Here, for the first time, we introduce a large‐scale 32 × 32 image sensor array based on high performance MSM *a*‐Ga_2_O_3_ PDs on a Si/SiO_2_ substrate. The Ga_2_O_3_ PDs exhibit ultrahigh performances, including a super‐high specific detectivity (D*) of 3.9 × 10^16^ Jones, giant responsivity (*R*) of 733 A W^−1^, and large photo‐to‐dark current ratio (PDCR) of 3.9 × 10^7^. Based on these highly sensitive PDs, solar‐blind imaging with high contrast has been obtained, taking advantage of the highly sensitive and uniform pixels in the image sensor array.

## 2 Results and Discussion

**Figure 1:**
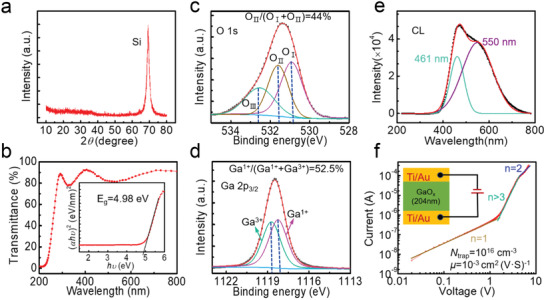
a) XRD pattern and b) transmittance spectrum of the annealed *a*‐Ga_2_O_3_ film. The inset in (b) is Tauc plot. XPS c) O 1s and d) Ga 2p_3/2_ spectrum of the annealed *a*‐Ga_2_O_3_ film. e) CL spectrum of the annealed *a*‐Ga_2_O_3_ film. f) Dark current–voltage characteristics of the annealed *a*‐Ga_2_O_3_ film and the corresponding fitting curves with the SCLC model. The inset in (f) presents the schematic diagram of the SCLC measurement.

Figure S1, Supporting Information, shows the device structure diagram. After the Ti/Au interdigital electrodes were formed, the device was post‐annealed (PA) in nitrogen atmosphere. Experimental details are given in the Experimental Section. According to the result of X‐ray diffraction (XRD) pattern in **Figure** **1**a, the annealed Ga_2_O_3_ film is evidently amorphous. As shown in the UV–visible light transmittance in Figure 1b, the annealed *a*‐Ga_2_O_3_ film has strong light absorption in solar‐blind band. Optical band gap is extracted to be 4.98 eV through the Tauc method. Scanning electron microscopy (SEM) and atomic force microscopy (AFM) are adopted to obtain surface morphology information of the annealed *a*‐Ga_2_O_3_ film. As exhibited in Figure S2, Supporting Information, the surface is smooth with root‐mean‐square surface roughness of 1.67 nm. Obvious nanoparticulate morphology can be identified from the SEM surface morphology image in size of tens of nanometers. In brief, the annealed *a*‐Ga_2_O_3_ film grown by magnetron sputtering has a smooth surface morphology with high uniformity, which lays a solid foundation for large‐scale array preparation.

X‐ray photoelectron spectroscopy (XPS) was utilized to characterize the composition information of annealed *a*‐Ga_2_O_3_ film. Figure 1c shows the corresponding XPS O 1s core‐level spectrum. O_І_, O_ІІ_, and O_ІІІ_ correspond to metal‐oxide bond, oxygen vacancy‐related bond, and surface hydroxide‐related bond, respectively, and their corresponding peak area ratio was utilized to represent the ratio of each component.^[^ ^42^ ^]^ The intensity ratio was O_ІІ_/(O_І_+O_ІІ_) = 44%, which reveals the presence of high concentration oxygen vacancies (*V*_O_′s) in the annealed *a*‐Ga_2_O_3_ film. In the Ga 2p_3/2_ spectrum, two components of Ga^1+^ and Ga^3+^ are associated with Ga_2_O and Ga_2_O_3_, respectively. The percentage of Ga_2_O species in the annealed *a*‐Ga_2_O_3_ film is as high as Ga^1+^/(Ga^1+^+Ga^3+^) = 52.5%, which confirms the O‐deficient situation. Based on the peak area ratios of O 1s and Ga 2p_3/2_, O/Ga ratio of the annealed *a*‐Ga_2_O_3_ is figured out to be ≈1, smaller than the stoichiometric ratio of 1.5. Energy dispersive X‐ray spectroscopy (EDS) has also been conducted to validate the chemical composition of annealed *a*‐Ga_2_O_3_, and statistic distribution of O/Ga ratio was investigated (Figure S3, Supporting Information). XPS study of the as‐deposited *a*‐Ga_2_O_3_ film was also analyzed, as presented in Figure S4a,b, Supporting Information. The ratio of *V*_O_‐related O_ІІ_ and the percentage of Ga_2_O species were 41% and 49.3%, respectively, suggesting that the annealing process results in an increase of the *V*_O_ concentration.

Figure 1e shows the cathodoluminescence (CL) spectrum of the annealed *a*‐Ga_2_O_3_ film. No band‐edge emission was observed, indicating the existence of massive compensating deep level traps. The CL spectrum exhibits broad blue emission with two peaks centered around 461 (2.7 eV) and 550 nm (2.3 eV) obtained by Gaussian fitting. The CL spectrum of as‐deposited *a*‐Ga_2_O_3_ film also shows a broad blue emission (Figure S4c, Supporting Information), but the emission peak red‐shifts with a much lower intensity compared to the annealed film. The blue emission is associated with the recombination process of electrons at donor state and holes at acceptor state via neutral defects, generally, the *V*_O_.^[^ ^43^ ^]^ Therefore, enhancement of blue emission of the annealed *a*‐Ga_2_O_3_ film may stem from the increase of *V*_O_ concentration after annealing, which can serve as effective carrier recombination centers.

A lower electron trap concentration and a higher carrier mobility are always more popular for Ga_2_O_3_ PDs. We quantitatively analyzed the change of electron trap density (*N*_trap_) and electron mobility (*μ*) of the *a*‐Ga_2_O_3_ film before and after annealing, using a space charge‐limited current (SCLC) model (Figure 1f). The parameters *N*_trap_ and *μ* are 1 × 10^16^ cm^−3^ and 1 × 10^−3^ cm^2^ V^−1^ s^−1^, respectively, based on the fitting method in the literatures.^[^ ^44^, ^45^ ^]^ On the other hand, as exhibited in Figure S4d, Supporting Information, the *N*_trap_ and *μ* values of the as‐deposited *a*‐Ga_2_O_3_ film are 4.5 × 10^16^ cm^−3^ and 4.5 × 10^−4^ cm^2^ V^−1^ s^−1^, respectively. Hence, the annealed *a*‐Ga_2_O_3_ film is significantly better in photosensor application for its lower *N*_trap_ and higher *μ*.

**Figure 2:**
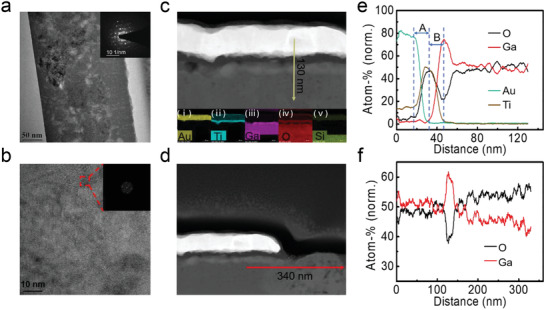
a) Cross‐sectional TEM image of the annealed *a*‐Ga_2_O_3_ film in the area without Ti/Au electrodes covered. The inset shows the selected‐area diffraction pattern. b) High‐resolution TEM image of the annealed *a*‐Ga_2_O_3_ film. The inset is FFT diffraction pattern of the selected region in red dashed box. Cross‐sectional HAADF‐STEM image of c) the PA *a*‐Ga_2_O_3_ SBPD with the *a*‐Ga_2_O_3_ film fully covered with Ti/Au electrodes (insets i–v show the EDS analysis of the device from top to bottom) and d) with the *a*‐Ga_2_O_3_ film partly covered with Ti/Au electrodes. EDS data of atomic ratio of e) the annealed *a*‐Ga_2_O_3_ film along the yellow arrow line in (c) from top to bottom, and (f) along the red arrow line in (d) from left to right.

To better understand the lower concentration of *N*_trap_ and higher *μ* of the annealed *a*‐Ga_2_O_3_ film, transmission electron microscopy (TEM) was used to figure out the microstructure of *a*‐Ga_2_O_3_. As presented in **Figure** **2**a, the annealed *a*‐Ga_2_O_3_ film presents obvious local crystallization, which can be supported by the selected‐area diffraction pattern (Figure 2a inset), and further corroborated by the high‐resolution TEM image and the corresponding fast Fourier transformed (FFT) diffraction pattern with clear diffraction spot (Figure 2b inset). Orderly oriented crystallites on the order of a few nanometers are found to exist in the annealed *a*‐Ga_2_O_3_ film. Therefore, the annealed *a*‐Ga_2_O_3_ film is actually a mixture of amorphous and crystalline Ga_2_O_3_. There may exist a grain boundary at the interface of different crystallization areas with different orientations and amorphous/crystalline phase junction in the Ga_2_O_3_ film. Space‐charge region may emerge around these regions, which can enhance the photogenerated carrier separation and transport efficiency.^[^ ^46^, ^47^ ^]^ However, no crystallites are observed in the as‐deposited Ga_2_O_3_ film (Figure S5a,b, Supporting Information), where no sharp diffraction spot is observed in its FFT diffraction pattern. Hence, we conclude that the PA process brings about partial crystallization and thus improves the film quality with higher *μ* and lower *N*_trap_.

Figure 2c exhibits cross‐sectional high‐angle annular dark‐field scanning transmission electron microscopy (HAADF‐STEM) image of the PA *a*‐Ga_2_O_3_ covered by Ti/Au electrodes. Insets i–v show its corresponding element distribution, which clearly demonstrates the device structure of the MSM *a*‐Ga_2_O_3_ SBPD. As presented in Figure 2e, by doing line scan EDS analysis, we obtain the atomic ratio distribution of the device along the yellow arrow line in Figure 2c. The atomic ratio of oxygen decreases abruptly at the interface between Ti and Ga_2_O_3_ (interval B), because oxygen was taken away from Ga_2_O_3_ by Ti oxidation during annealing. This is consistent with the existence of oxygen in the Ti layer (interval A in Figure 2e, inset iv in Figure 2c). Yet, the as‐fabricated MSM *a*‐Ga_2_O_3_ SBPD shows no obvious decrease of oxygen atomic ratio at the interface of *a*‐Ga_2_O_3_ and Ti layer, as shown in Figure S5c,e, Supporting Information. This result indicates that the PA process might introduce *V*_O_s at Ti/Ga_2_O_3_ interface. In the cross‐sectional HAADF‐STEM image in Figure 2d, the annealed *a*‐Ga_2_O_3_ film is partly covered by Ti/Au electrodes. Line scan of EDS along the red arrow line was also performed, with the results shown in Figure 2f. The oxygen atomic ratio in the region covered by Ti/Au electrodes is lower than that in the bare Ga_2_O_3_ region, thus the Ti/Au capping tends to cause oxygen deficiency in the electrode coverage region. This phenomenon was also observed in the as‐fabricated MSM *a*‐Ga_2_O_3_ SBPD (Figure S5d,f, Supporting Information). To conclude, there exist more *V*_O_s at Ti/*a*‐Ga_2_O_3_ interface than that in the uncovered region, and this trend becomes more obvious after PA process.

**Figure 3:**
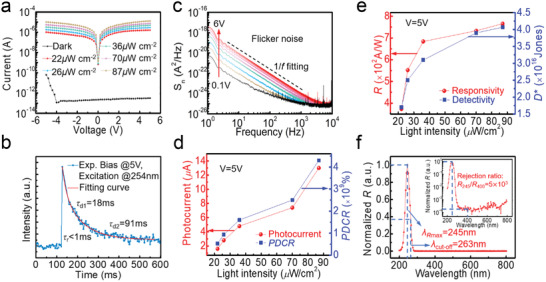
a) Semi‐log current–voltage characteristics of the PA *a*‐Ga_2_O_3_ PD in the dark and under excitation of different 254 nm light intensity. b) Transient photoresponse characteristic curve obtained by 10 ms 254 nm pulsed light excitation at 5 V. c) Noise spectral power density of the PD at various bias voltages in the dark. Relationship between d) photocurrent, PDCR, and e) *R*, *D* ^∗^ with light intensities. f) Wavelength‐dependent photoresponse of the PDs at 5 V. The inset is semi‐log normalized *R* versus wavelength of *a*‐Ga_2_O_3_ PD at 5 V.

The photodetection characteristics of the PA *a*‐Ga_2_O_3_ SBPD were studied using 254 nm deep ultraviolet light. **Figure** **3**a presents the semi‐log current–voltage (*I–V*) characteristics of the PA *a*‐Ga_2_O_3_ PD in the dark and excited by different 254 nm light intensity. Dark current reached as low as 0.3 pA, and large photocurrent exceeding 10 µA was obtained at 5 V when PD was irradiated by 70 µW cm^−2^ light. Photocurrent increases with light intensity and an extremely high PDCR of 3.9 × 10^7^ was achieved at 5 V bias according to the equation

(1) $$PDCR\;=\frac{I_{\mathrm{photo}}-I_{\mathrm{dark}}}{I_{\mathrm{dark}}}$$

where *I*_photo_ and *I*_dark_ are photocurrent and dark current, respectively. Figure S6a, Supporting Information, presents current versus time response characteristics when the light was repeatedly turned on and off. Owing to higher electric field at higher voltage, more carriers could be collected and photocurrent was observed to increase with voltages. As shown in Figure 3b, transient photoresponse were conducted by 254 nm pulsed light excitation at 5 V. The rise time is less than 1 ms. The decay process can be well fitted with the equation:^[^ ^48^ ^]^

(2) $$I\;=I_{0}\;+Ae^{-t/\tau_{d1}}+Be^{-t/\tau_{d2}}$$

where *I*_0_, *t*, *A*, and *B* denote the steady‐state photocurrent, time, and constants.*τ*_d1_ and *τ*_d2_ denote the fast and slow relaxation time constants, respectively.^[^ ^49^ ^]^ *τ*_d1_/*τ*_d2_ for the PDs are derived to be 18/91 ms.

The PA *a*‐Ga_2_O_3_ SBPD was quantitatively assessed by some key figure‐of‐merits, including *R*, D* and external quantum efficiency (EQE). According to the equation:

(3) $$R\;=\frac{I_{\mathrm{photo}}-I_{\mathrm{dark}}}{P_{\lambda }S}$$

where *P_*λ*_* and *S* denote the light intensities and effective area of PDs, *R* is as high as 733 A W^−1^ at 5 V when *P_*λ*_* = 70 µW cm^−2^. Therefore, an extremely high gain‐bandwidth product of *R*/*τ* >10^4^ has been achieved, signifying a remarkably high overall performance of the device. The most basic performance metric of PDs, that is, sensitivity, is traditionally exemplified by noise equivalent power (NEP). Generally, the D* is more recommended to assess the ability of sensing weak signal of PDs with different sizes and bandwidths. Based on the measured *R* and noise spectral density in Figure 3c,^[^ ^50^ ^]^ a super‐high D* of 3.9 × 10^16^ Jones is realized at 5 V at 1 kHz according to the relations:

(4) $$D^{*}=\frac{\sqrt{SB}}{NEP}$$

(5) $$NEP\;=\frac{i_{n}}{R}$$

where *B* and *i*_n_ denote the bandwidth and noise current, respectively. Noise equivalent power (NEP) was estimated to be as low as 3.5 fW Hz^−1/2^, indicating that this PD can detect the 254 nm light with intensity down to 18 pW cm^−2^. This suggests the strong capability of the device to provide an ultrahigh signal‐to‐noise ratio with respect to the noise current. As shown in Figure 3c, 1/*f* noise dominates the noise of this PD in a wide frequency range. The noise power density of the as‐fabricated *a*‐Ga_2_O_3_ SBPD (Figure S6a, Supporting Information) follows the same trend but with larger noise. Since 1/*f* noise generally stems from the trapping and detrapping processes of carriers, the suppression of 1/*f* noise in PA *a*‐Ga_2_O_3_ SBPD suggests that the annealing process passivates the electron traps of *a*‐Ga_2_O_3_ film, in accordance with the SCLC results. EQE is determined by:

(6) $$EQE\;=\frac{hc}{q\lambda }\;R\times 10^{2}\%$$

where h, *c*, and *λ* are Plank's constant, light velocity, and wavelength, respectively. The EQE can reach as high as 4.1 × 10^5^% in our device.

Then, these figure‐of‐merits under different light intensities were derived, as shown in Figure 3d,e. The photocurrent and PDCR increase almost linearly with the light intensities. *R* and D* are first enhanced with light intensities. However, *R* and D* increase slowly under higher light intensities, due to light absorption saturation or the complete filling of gain related defect states under higher light intensities.^[^ ^51^ ^]^ Figure 3f shows wavelength‐dependent photoresponse of the SBPD at 5 V in linear coordinates. The device reaches its maximum responsivity and cut‐off wavelength at 245 and 263 nm, respectively. The inset of Figure 3f presents the semi‐log plot of the normalized *R* versus wavelength. The UV/visible responsivity rejection ratio (*R*_245 nm_/*R*_400 nm_) is 5 × 10^3^. Photoelectric properties of the as‐fabricated *a*‐Ga_2_O_3_ SBPD were also measured, as presented in Figure S6 and Table S1, Supporting Information. Obviously, the performance of PA *a*‐Ga_2_O_3_ SBPD shows great superiority compared with the as‐fabricated one and most of the previously reported Ga_2_O_3_ PDs (Table S2, Supporting Information). Naturally, it is notably necessary to figure out the underlying mechanism behind the excellent photodetection performance of the *a*‐Ga_2_O_3_ SBPD by post‐annealing process.

The ultrahigh *R* and gain‐bandwidth product imply the presence of internal gain mechanism in PA *a*‐Ga_2_O_3_ SBPD. Gain mechanism has also been reported in GaN MSM PDs.^[^ ^52^, ^53^ ^]^ Most likely, the increased electron injection from the metal contact should be responsible for the gain mechanism due to the barrier height lowering effect. This is caused by photoinduced hole accumulation, which may originate either from the trapping of holes close to the metal contact or the mobility difference between electrons and holes.^[^ ^54^ ^]^ In particular, electrons drift faster than holes, thus leaving a residual density of holes near the contact. For Ga_2_O_3_, the polaron mobility^[^ ^55^ ^]^ was estimated to be *μ*≈10^−6^ cm^2^ V^−1^ s^−1^ at room temperature.^[^ ^54^, ^56^ ^]^ Such low mobility suggests that electrons may circulate many times in the channel during the lifetime of a photogenerated electron‐hole pair. Photoexcited holes are probably captured by acceptor‐like defects near metal/Ga_2_O_3_ interface and body area, leading to the barrier height lowering effect and accordingly the internal gain enhancement.^[^ ^52^ ^]^

**Figure 4:**
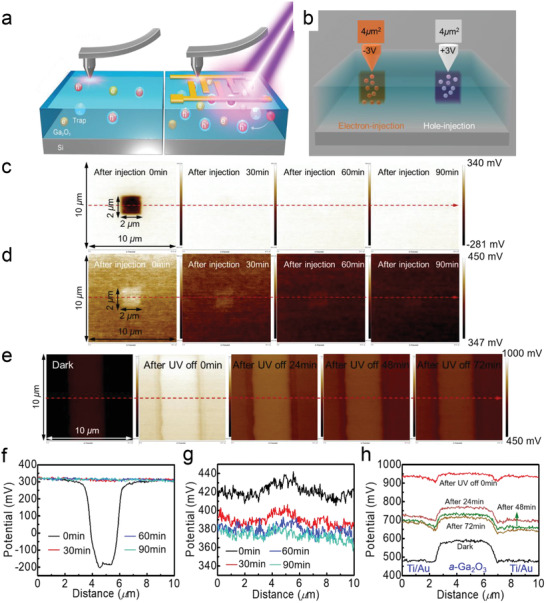
a) Schematics of the in situ KPFM electrical nanotechnology of *a*‐Ga_2_O_3_ film and mechanism of surface potential variation in the dark and light excitation. The scanning area is 10 µm × 10 µm. b) Schematic of charge injection to *a*‐Ga_2_O_3_ film. Snapshots of surface potential variation for c) the trapped electrons and d) holes at 0, 30, 60, and 90 min. e) Surface potential variation process in a 10 µm × 10 µm scanning area of the PA *a*‐Ga_2_O_3_ SBPD before and after 254 nm light illumination. f–h) Surface potential variation process along the red arrow line in Figures 4c,d,e, respectively. Note that all color bars are normalized to a uniform scale.

To confirm this hypothesis experimentally, Kelvin probe force microscopy (KPFM) on the PA *a*‐Ga_2_O_3_ SBPD was performed, as shown in **Figure** **4**a. First, the capability of electron and hole to be trapped was characterized separately. As illustrated in Figure 4b, two 10 × 10 µm^2^ areas were scanned, in which a 2 × 2 µm^2^ area was scanned for electron and hole injections at −3 and +3 V, respectively. The dynamic variation process of electron/hole diffusion was recorded, and the mapping of the surface potential at different times is presented in Figure 4c,d for electrons and holes injection, respectively. The electron‐injection area demonstrates a trend of quicker carrier vanishing, indicating a faster diffusion rate of the electrons in Ga_2_O_3_. Surface contact potential difference (CPD) after the charge injection along the red dashed line in Figure 4c,d was derived, as plotted in Figure 4f,g, respectively. Compared to the state when the carriers were just injected, the CPD exhibited only about 58% decay after 30 min for the hole‐injection area. However, in electron‐injection region, the surface potential totally returned to its original value after 30 min. Consequently, the KPFM results lend strong support to the viewpoint that holes are more easily trapped in the PA *a*‐Ga_2_O_3_ film.

Subsequently, in situ KPFM of the PA *a*‐Ga_2_O_3_ SBPD before and after light irradiation of 254 nm was performed. AFM image of scanning area is shown in Figure S7a, Supporting Information. The 10 × 10 µm^2^ scanning area includes part of the interdigital electrodes and the *a*‐Ga_2_O_3_ channel between the electrodes. First, CPDs of the scanning area in the dark and just after illuminated by 254 nm light were investigated. Then, dynamic variation processes of the CPD after turning the light off for 24, 48, and 72 min in the scanning area were recorded. The obtained surface potential distribution is shown in Figure 4e. Along the red arrow line in Figure 4e, the dynamic process of surface CPD was derived, as shown in Figure 4h. In the dark, the surface potential of PA *a*‐Ga_2_O_3_ channel is ≈100 mV higher than that of the Ti/Au metal stacks. As the device was subjected to light irradiation, the CPD in the scanning area shows an overall rise. Generally, increase in surface potential stems from the accumulation of holes or the loss of electrons.^[^ ^57^ ^]^ As stated above, light illumination can trigger generation of photocarriers in the *a*‐Ga_2_O_3_ film. The photo‐generated electrons can easily escape, thus leaving behind photo‐excited holes in the valence band or trapped in defect states, which increases surface CPD. It is worth noting that the surface CPD between Ti/Au metal stacks and PA *a*‐Ga_2_O_3_ channel is nearly negligible when the device was irradiated with light. This confirms the Schottky barrier lowering effect as a result of many acceptor‐like defect states at the interface between Ti and PA *a*‐Ga_2_O_3_.^[^ ^52^ ^]^ These acceptor‐like defect states are probably oxygen vacancies, as supported by the TEM and EDS results. When the light was turned off, the surface CPD of the scanning area decays abruptly at first, and then decreases slowly, which is consistent with the decay process of time‐dependent characteristics of this device with both fast and slow decay components.

As a comparison, we note that the as‐fabricated *a*‐Ga_2_O_3_ SBPD shows few CPD changes in the Ga_2_O_3_ channel after irradiated by light, while the Schottky barrier became higher. Therefore, with enhanced Schottky barrier lowering effect, the PA *a*‐Ga_2_O_3_ SBPD shows larger internal gain due to higher *V*_O_ concentration at the electrodes/PA *a*‐Ga_2_O_3_ interface. Besides, the *V*_O_s also play a key role in facilitating carrier recombination for faster response speed.^[^ ^58^, ^59^ ^]^ The details as well as energy band information are discussed in Figure S7–S9 and Notes S1 and S2, Supporting Information.

For the purpose of further examining the effect of PA process on *a*‐Ga_2_O_3_ SBPD, density functional theory (DFT) calculations were performed. Two kinds of *V*_O_′s were found to possibly exist in Ga_2_O_3_, namely, neutral*V*_O_ and +2 charged oxygen vacancy (*V*_O_^2+^). The generation of *V*_O_ will result in formation of defect levels in the bandgap, lying at 2.766, 2.695, and 2.030 eV above the valence band maximum (VBM) for O1, O2, and O3 sites, respectively. High density of states of electrons is strongly localized on these defects (Figure S10c,d, Supporting Information). Photoexcited holes are likely to be captured by these acceptor‐like traps. When the *V*_O_ is ionized to *V*_O_^2+^, the introduced defect levels move toward the conduction band minimum (CBM), lying at 4.705, 4. 715, and 4.759 eV above the VBM for O1, O2, and O3 sites, respectively. This difference between *V*_O_ and *V*_O_^2+^ can be explained by structural deformation in the supercell of Ga_2_O_3_ after oxygen vacancy relaxation, as shown in Figure S10h,i, Supporting Information. The effect of PA was simulated based on amorphous Ga_2_O_3_ by ab initio molecular dynamics. PA shifts the charge transfer level (*ε* (+2/0)) of *a*‐Ga_2_O_3_ toward the CBM direction, which means that *V*_O_^2+^ is easier to be created, contributing to the photocurrent. Another effect of annealing for Ga_2_O_3_ is structural reconstruction and diffusion. As shown in Figure S11d and Table S3, Supporting Information, the oxygen atoms surrounding oxygen vacancies are observed to move to occupy the original vacancy sites via reconstruction. On the other hand, Ga atoms tend to move away from oxygen vacancies, which are enhanced after annealing. Besides, *V*_O_ serves as an effective recombination center to promote photo‐excited carrier recombination. When a *V*_O_ traps a hole, it is transformed into *V*_O_^+^, while the defect level is located at ≈1.5 eV below the CBM. The *V*_O_^+^ will not donate electrons to the CBM but capture electrons from CBM or *V*_O_^2+^. Sequentially, recombination of electrons at CBM and holes at VBM via *V*_O_ occurs. With the increase of *V*_O_ concentration after annealing, more deeper defect levels emerge in the energy gap (Figure S12, Supporting Information). Consequently, the PA *a*‐Ga_2_O_3_ SBPD can achieve lower dark current, higher photocurrent as well as faster recovery speed. Ga vacancy (*V*_Ga_) in pure Ga_2_O_3_ or N‐doped Ga_2_O_3_ was also studied. The defect levels introduced by V_Ga_ are close to VBM, about 0.18 eV above VBM for pure Ga_2_O_3_ and 0.41 eV above VBM for N‐doped Ga_2_O_3_. They are unlikely to act as recombination center. Oxygen vacancy plays a leading role in the device performance. Detailed discussions are shown in Notes S3–S6, Supporting Information.

**Figure 5:**
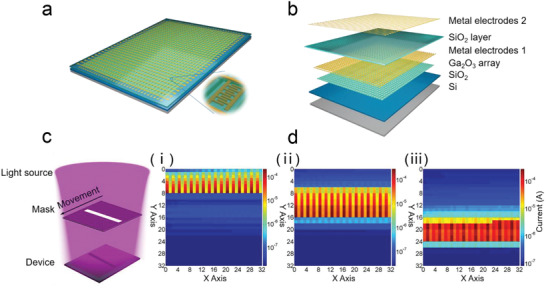
a) Schematic illustration of the fabricated 32 × 32 *a*‐Ga_2_O_3_ image sensor. Inset is the enlarged picture of a single pixel in array. b) Exploded schematic of the image sensor. c) Schematic diagram of the imaging operation for light beam movement measurement. d) Demonstration of the output image of the light beam at different positions during the movement process.

The supreme performance of the PA *a*‐Ga_2_O_3_ SBPDs promises their tremendous application potential in image sensors. To verify this prospect, the SBPDs were integrated and expanded to a large‐scale 32 × 32 image sensor array, as schematically shown in **Figure** **5**a. The magnified diagram of one pixel is also exhibited in Figure 5a. In the image sensor array, each pixel was isolated via wet etching of the *a*‐Ga_2_O_3_ layer with phosphoric acid (H_3_PO_4_). The size of each pixel is 400 µm × 440 µm with a spacing of 180 µm. Deconstructed graph of the *a*‐Ga_2_O_3_ array is demonstrated separately in Figure 5b. Details of fabrication process of the image sensor array can be found in the Experimental Section.

After preparation, the array was packaged and connected to the self‐designed printed circuit board. To demonstrate the imaging performance, the packaged chip was assembled on a readout circuit module and tested in our home‐made imaging system (see Figure S15a,b, Supporting Information). The read‐out circuit diagram of the imaging system is shown in Figure S16, Supporting Information. For imaging application, high uniformity is required for both dark and photo‐current in all the pixels, for the sake of high resolution and contrast. For the uniformity test, each pixel in the array was evaluated by measuring its dark and photo‐current. The details are shown in Figures S17, S18 and Note S7, Supporting Information. The image sensor array demonstrates satisfactory uniformity of dark current, whereas the photo‐current displays relatively large fluctuation. This is because of the uneven light distribution during measurement, as well as the cross‐talk effect between neighboring pixels. As displayed in Figure 5c, the imaging functionality was eventually demonstrated by recognizing the moving objects projected onto the image sensor array. The photocurrent distribution was utilized to signify the optical image. As presented in Figure 5di–iii, the images of the projected light beam at various positions during the movement process were recorded. 3D photocurrent distribution of all pixels at 4 V is presented in Figure S19, Supporting Information. The image sensor array shows high contrast of irradiated and unirradiated regions, and the light beam in the moving process is clearly presented. Compared with the single device, we obtained relatively higher photo‐ and dark current of the pixels in the array, and blurred edges were also recorded for the array device. These phenomena are probably due to the unsuppressed sneak current between neighboring irradiated and unirradiated pixels. Such cross‐talk issue can be potentially mitigated by connecting a switching transistor in series to each pixel in the array. The present image shows outstanding recognition capability, validating the application potential of Ga_2_O_3_ PDs in future solar‐blind imaging technology for display and tracking applications.

## 3 Conclusion

In summary, we have demonstrated superhigh performance MSM *a*‐Ga_2_O_3_ solar‐blind photodetectors by an innovative post‐annealing process. The photodetector exhibits superior sensitivity in solar‐blind region including extremely high *R* of 733 A W^−1^, PDCR of 3.9 × 10^7^, and super‐high D* of 3.9 × 10^16^ Jones. Thanks to the improved film quality by post‐annealing, including higher electron mobility, lower electron trap density, and enhanced photoexcited carrier recombination, extremely low dark current of 0.3 pA and short decay time of *τ*_r_/*τ*_d1_ = 1/18 ms at 5 V bias are achieved in our device. The lowering of barrier height accounts for the huge internal gain of the photodetectors. In this process, the oxygen vacancies make a contribution to the internal gain and promote the electron‐hole recombination process for fast recovery of the photodetectors. Remarkably, for the first time, we report a large‐scale, high‐uniformity 32 × 32 image sensor array based on the high‐performance PA MSM *a*‐Ga_2_O_3_ SBPDs, which presents excellent optical pattern recognition capability. This work may pave a way toward large‐scale and high‐resolution Ga_2_O_3_ optoelectronic device integration for applications in optical communication, digital display, artificial intelligence retina, and so forth.

## 4 Experimental Section

### Deposition of Ga_2_O_3_ Film and Fabrication of Single PD

Ga_2_O_3_ film was deposited by sputtering Ga_2_O_3_ target at room temperature. The sputtering power and pressure were 60 W and 0.4 Pa, respectively. The deposition atmosphere was 0.3 sccm oxygen and 30 sccm argon. The fabrication process and size of device follows a previous report.^[^ ^60^ ^]^ Then, the post‐annealing process of the device was conducted under 400 °C in N_2_ ambient for 10 min with no bombardment by Plasma Enhanced CVD (PECVD) furnace tube. The pressure was 100 Pa. It takes 20 min to heat up to 400 °C, and keep it at 400 °C for 10 min, after 20 min to cool down to 100 °C.

### KPFM Measurement

Bruker Multi Mode 8 AFM was used for the KPFM measurement with combination of contact mode and KPFM based on amplitude modulation of tapping mode using a Pt/Ir conductive tip. The scan rate was 1 Hz. The sample was glued to the support with conductive silver glue. First, the surface topography of the sample was obtained by tapping mode to confirm the surface was smooth. Then, lift the probe to a certain height, apply a voltage to the probe, the sample was biased at 0 V, use the topography information obtained from the first scan to keep the vertical distance between the probe and the sample constant, and then inject electrons or holes in an area of 2 µm × 2 µm. The tip‐sample surface distance was 80 nm in the KPFM measurement. Next, switching to tapping mode to measure the surface potential of the scanning area at different time points. For the in situ KPFM of the PA *a*‐Ga_2_O_3_ SBPD after light irradiation of 254 nm, the only difference was illuminating the sample with an external light source during the KPFM measurement. After 254 nm light illumination, the change of surface potential of the sample was recorded at different time points.

### Image Sensor Array Fabrication Process

After the preparation of Ga_2_O_3_ film, device isolation was conducted by wet‐etching with phosphoric acid. The metal electrodes 1 with all the interdigital electrodes was deposited by electron beam evaporation. The thickness of first electrode layer (Ti/Au metal stack) was 10/50 nm. After that, a SiO_2_ insulating layer was deposited by PECVD, followed by wet‐etching in order to expose an area for collecting second layer metal electrodes and first layer metal electrodes. Finally, the metal electrodes 2 (Ti/Au = 20/100 nm) was deposited. Similarly, the post‐annealing process was conducted in CVD furnace tube. To facilitate bonding, an additional metal layer of Ti/Au = 5/20 nm was deposited on all the pads around. So far, a 32 × 32 image sensor array with total area of 20 mm × 23 mm was successfully fabricated.

### Material Characterization and Photoelectric Measurement

The film quality was measured by XRD with scanning from 10° to 80° (Bruker D8 focus). The transmittance of the Ga_2_O_3_ film was characterized by UV–VIS–NIR spectrophotometer (Agilent Cary 7000). The elemental composition and analysis were carried out with XPS (ESCALAB 250Xi). The film thickness and surface were characterized by SEM (Zeiss Supra 55). AFM were utilized to investigate the surface topography and potential (Bruker Multi Mode 8). The photoelectric properties were tested on a semiconductor parameter analyzer (4200SCS, Keithley). The light source was well calibrated before test (Tanon UV‐100, LH‐126C). The wavelength‐dependent photoresponse was conducted by a spectra measurement system (Zolix DSR‐OS‐X150A‐ZKDDZ).

## Conflict of Interest

The authors declare no conflict of interest.

## Supporting information

Supporting Information

## Data Availability

The data that support the findings of this study are available from the corresponding author upon reasonable request.
