# Supplementary material for: Ultra‐High Performance Amorphous Ga2O3 Photodetector Arrays for Solar‐Blind Imaging
Source: Adv Sci (Weinh). 2021 Aug 13;8(20):2101106. doi: 10.1002/advs.202101106 (PMC8529488; doi:10.1002/advs.202101106)
Supplement: Supplementary file 1 — Supporting Information [file ADVS-8-2101106-s001.pdf]

© 2021 Wiley-VCH GmbH

ADVANCED SCIENCE
Open Access

Supporting Information

for *Adv. Sci.,* DOI: 10.1002/advs.202101106

Ultra-High Performance Amorphous Ga_2O_3 Photodetector Arrays for Solar-Blind Imaging

Yuan Qin, Li-Heng Li, Zhaoan Yu, Feihong Wu, Danian Dong, Wei Guo, Zhongfang Zhang, Jun-Hui Yuan, Kan-Hao Xue, Xiangshui Miao, Shibing Long**

Supporting Information

Ultra-High Performance Amorphous Ga_2O_3 Photodetector Arrays for Solar-Blind Imaging

Yuan Qin#, Li-Heng Li#, Zhaoan Yu, Feihong Wu, Danian Dong, Wei Guo, Zhongfang Zhang, Jun-Hui Yuan, Kan-Hao Xue, Xiangshui Miao, Shibing Long**

Y. Qin, D. Dong, Dr. Z. Yu
Key Laboratory of Microelectronics Devices & Integration Technology
Institute of Microelectronics of Chinese Academy of Sciences
Beijing 100029, China

Y. Qin, F. Wu, W. Guo, Z. Zhang, Prof. S. Long (shibinglong@ustc.edu.cn)
School of Microelectronics
University of Science and Technology of China
Hefei, Anhui 230026, China

L. Li, J. Yuan, Prof. K. Xue (xkh@hust.edu.cn), Prof. X. Miao
Wuhan National Laboratory for Optoelectronics, School of Optical and Electronic Information
Huazhong University of Science and Technology
Wuhan 430074, China

#These authors contributed equally to this work.

The supporting information file includes:

Figure S1. Schematic diagram of the MSM *a*-Ga_2O_3 SBPD.

Figure S2. AFM surface morphology image and SEM image of the annealed *a*-Ga_2O_3 film.

Figure S3. Statistic distribution of the value of O/Ga ratio based on EDS element analysis of the annealed *a*-Ga_2O_3 film.

Figure S4. XPS analysis, CL spectrum and dark current-voltage characteristics of the as-deposited *a*-Ga_2O_3 thin film based on SCLC model.

Figure S5. TEM results analysis of the as-deposited *a*-Ga_2O_3 thin film.

Figure S6. Photoelectric characteristics of the as-fabricated MSM *a*-Ga_2O_3 SBPD.

Figure S7. KPFM results of as-fabricated MSM a-Ga2O3 SBPD and work function measurement.

Figure S8. Energy band information of as-deposited and post-annealed *a*-Ga_2O_3 film.

Figure S9. PL spectrum of the as-deposited and post-annealed *a*-Ga_2O_3 film.

Figures S10/S11/S12. DFT calculation analysis on Ga_2O_3.

Figure S13. Calculated density of states for N-doped Ga_2O_3 and its corresponding energy band structure diagram.

Figure S14.Energy band structure diagrams and differential charge diagram of Ga_2O_3 and N-doped Ga_2O_3 with V_{Ga}.

Figure S15. Schematic illustration of the homemade 32×32 array imaging system.

Figure S16. Circuit diagram of the read-out circuits.

Figure S17. Dark current and photocurrent uniformity of the 32×32 *a*-Ga_2O_3 image sensor array.

Figure S18. Statistical distribution of I_{photo}/I_{dark} ratio of the array under 4 V bias voltage.

Figure S19. Three-dimension photocurrent distribution of all pixels in different position in the movement process.

Table S1. List of key figures-of-merit of the as-fabricated MSM *a*-Ga_2O_3 SBPD.

Table S2. Comparison between previously reported Ga_2O_3 PDs and our post-annealed MSM *a*-Ga_2O_3 SBPDs.

Table S3. The distance variations between the oxygen vacancy position and the neighboring Ga atoms before and after annealing of the amorphous Ga_2O_3 film.

Supplementary Note 1. KPFM results of as-fabricated MSM *a*-Ga_2O_3 SBPD and work function calculation.

Supplementary Note 2. Energy band information of as-deposited and post-annealed *a*-Ga_2O_3 film.

Supplementary Note 3. DFT calculations on Ga_2O_3 and the corresponding analysis.

Supplementary Note 4. Discussion of recombination model.

Supplementary Note 5. Discussion of Ga vacancy.

Supplementary Note6. Computational details of DFT calculation.

Supplementary Note 7. Evaluation of uniformity test of the image sensor array.

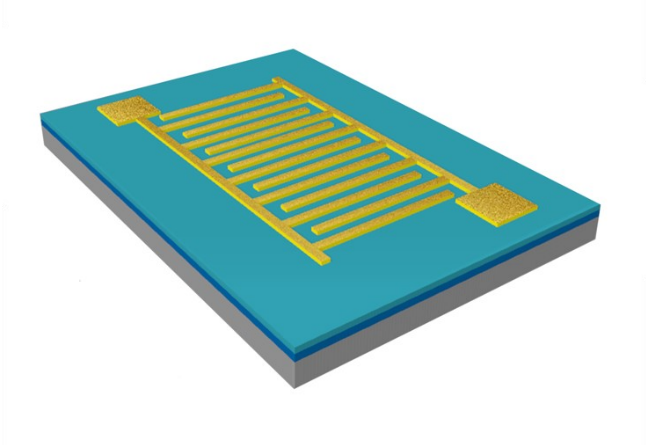

Figure S1. Schematic diagram of the MSM a-Ga_2O_3 SBPD.

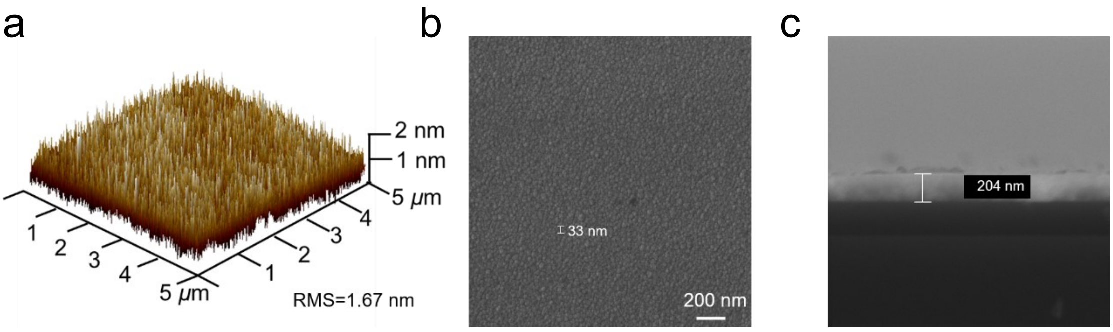

Figure S2. a) 3D-AFM surface morphology image in a 5×5 μm^2 scanning area. b) Top-view and c) cross-section SEM image of the annealed a-Ga_2O_3 film.

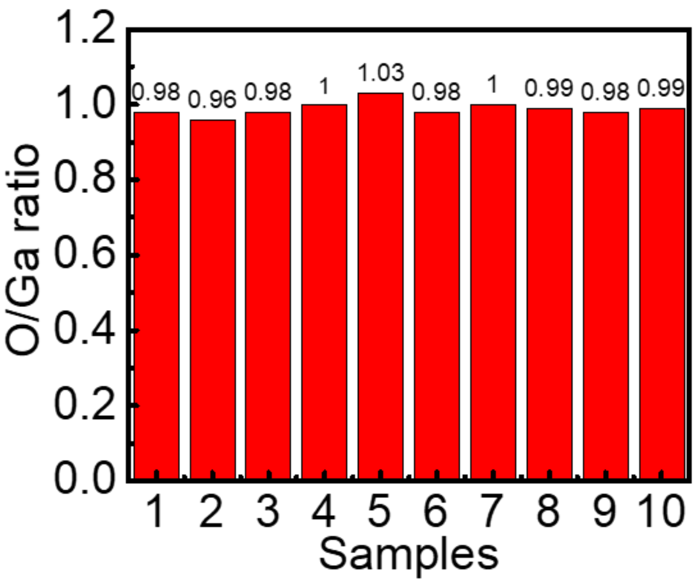

Figure S3. Statistic distribution of the value of O/Ga ratio based on EDS element analysis of the annealed *a*-Ga_2O_3 film. EDS analysis shows that the O/Ga ratio varies from 0.96 to 1.03 with a small fluctuation, suggesting the good reproducibility of film growth process.

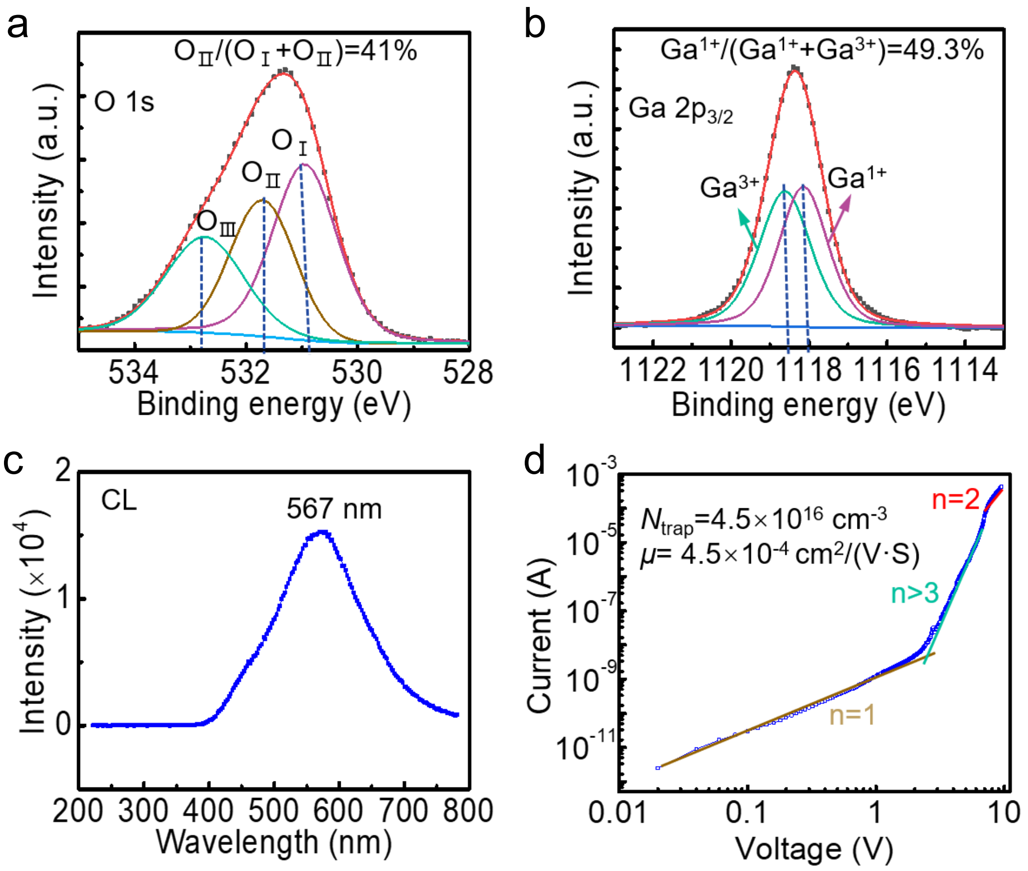

Figure S4. XPS a) O 1s and b) Ga $2p_{3/2}$ core-level spectrum of the as-deposited *a*-Ga_2O_3 thin film. c) Cathodoluminescence spectrum of the as-deposited *a*-Ga_2O_3 thin film with a broad defect peak centered around 567 nm. d) Dark current-voltage characteristics of the as-deposited *a*-Ga_2O_3 thin film and the corresponding fitting curves with the SCLC model.

Based on XPS results, the ratio of V_O related O_{II} was estimated to be 41% for the as-deposited *a*-Ga_2O_3 thin film, which is lower than that of annealed *a*-Ga_2O_3 thin film. This result also can be verified by the percentage of Ga_2O species of 49.3% in the as-deposited *a*-Ga_2O_3 film, suggesting that annealing process results in the increase in the concentration of V_O. As is shown in Figure S4c, the broad defect peak centered around 567 nm indicates the existence of V_O in the as-deposited *a*-Ga_2O_3 film. However, the CL spectrum intensity of the as-deposited *a*-Ga_2O_3 film is lower than that of annealed *a*-Ga_2O_3 film, further confirming that annealed *a*-Ga_2O_3 film has higher concentration of V_O. According to SCLC model, the electrical properties of annealed *a*-Ga_2O_3 film is clearly better with lower concentration of trap density and higher electron mobility, which is significantly better for photosensor application.

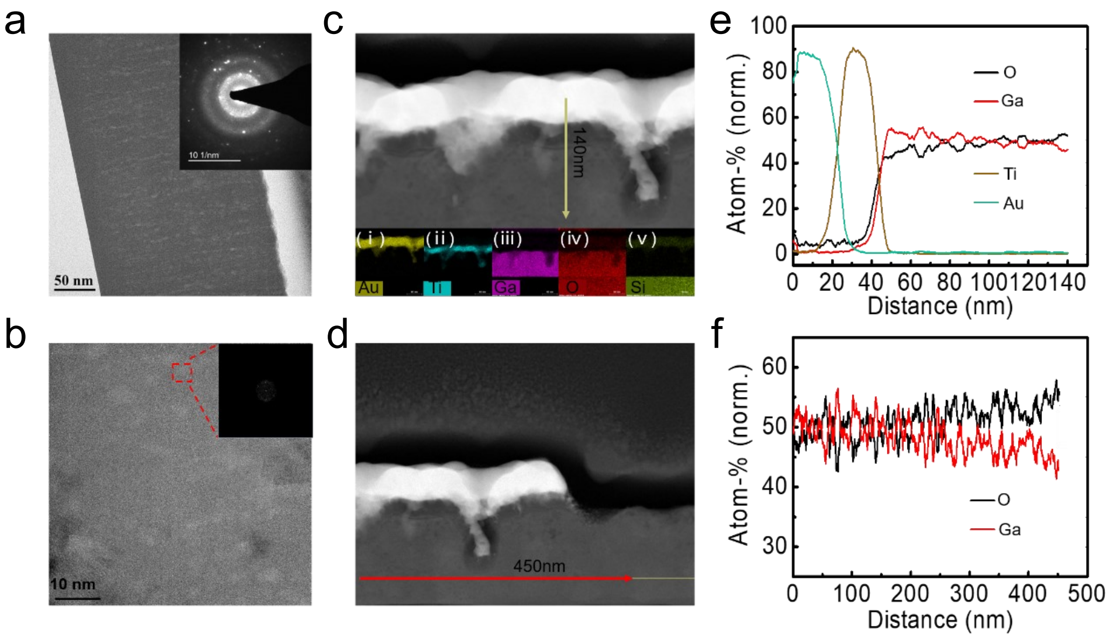

Figure S5. a) Cross-sectional TEM image of the as-deposited *a*-Ga_2O_3 film without Ti/Au electrodes. The inset shows the selected-area diffraction pattern of the film. b)

High-resolution TEM image of the unannealed *a*-Ga_2O_3 film. The inset is the FFT diffraction pattern of the selected region in red dashed box. c) Cross-sectional HAADF-STEM image of the as-fabricated *a*-Ga_2O_3 SBPD with Ti/Au electrodes. Insets i–v show the EDS analysis of the as-fabricated *a*-Ga_2O_3 SBPD with Ti/Au electrodes from top to bottom. d) Cross-sectional HAADF-STEM image of the as-fabricated *a*-Ga_2O_3 SBPD with the unannealed *a*-Ga_2O_3 film partly covered by Ti/Au electrodes. e) EDS data of atomic ratio of the unannealed *a*-Ga_2O_3 film along the yellow arrow line in c) from top to bottom. f) EDS data of atomic ratio of the unannealed *a*-Ga_2O_3 film along the red arrow line in d) from left to right.

From the cross-sectional TEM image of the unannealed *a*-Ga_2O_3 film, no obvious crystallization is observed in the as-deposited Ga_2O_3 film. The high-resolution TEM image shows that the atomic arrangement is totally disordered. There is no obvious diffraction spot appear in the selected-area diffraction pattern and FFT diffraction pattern of the as-deposited Ga_2O_3 film, suggesting the amorphous characteristic of the as-deposited Ga_2O_3 film. From the line scan EDS analysis in Figure S5e, it can be found that no obvious decrease of oxygen atomic ratio at the interface of *a*-Ga_2O_3 and Ti metal layer. This result suggests that the post annealing process can introduce oxygen vacancy at the interface of *a*-Ga_2O_3 and Ti. In Figure S5f, the trend of atomic ratio is similar to that of annealed Ga_2O_3 film, indicating there exist more oxygen vacancies at the interface of Ti metal layer and *a*-Ga_2O_3 than that in the uncovered area.

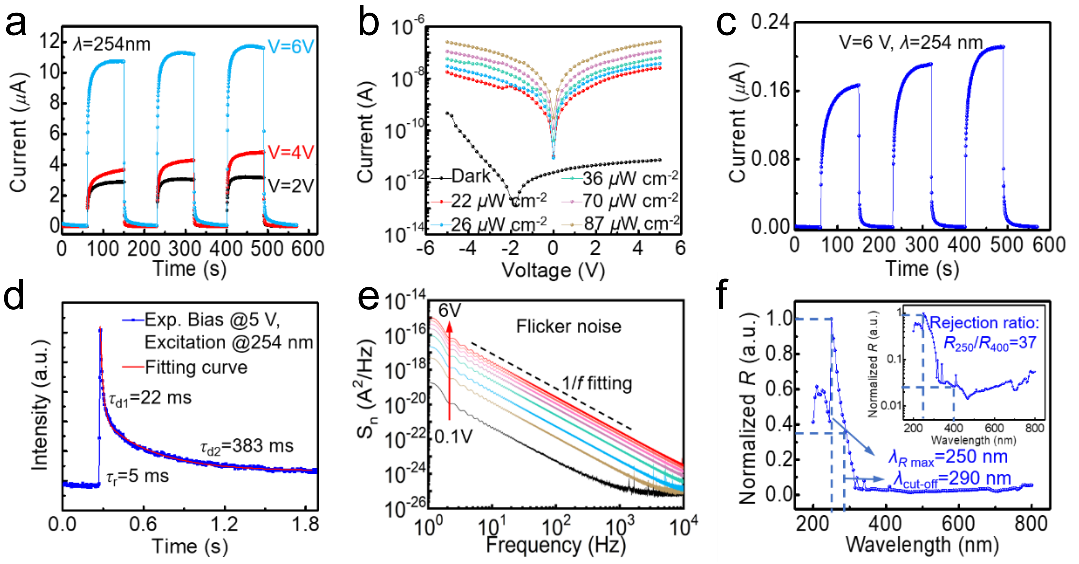

Figure S6. a) Time-dependent photoresponse characteristics of the PA MSM *a*-Ga_2O_3 SBPD at different bias voltages illuminated under 254 nm light with intensity of 70 μW/cm^2. b) Semi-log current−voltage characteristics of the as-fabricated MSM *a*-Ga_2O_3 SBPD in the dark and under 254 nm light illumination with different light intensities. c) Time-dependent photoresponse characteristics of the device at V=6 V illuminated under 254 nm light with intensity of 70 μW/cm^2. d) Transient photoresponse characteristic measured under excitation of 254 nm pulsed light at 5 V. e) Frequency-dependent noise power density of the as-fabricated MSM *a*-Ga_2O_3 SBPD at various bias voltages in the dark. f) Normalized spectral response of the as-fabricated MSM *a*-Ga_2O_3 SBPD at V=5 V. Inset shows semi-log normalized *R vs*. wavelength of the device at V= 5 V.

As shown in Figure S6b, it can be found that the current increases a lot when the device is under 254 nm light illumination, and the photocurrent increases with the light intensity. However, dark current of the as-fabricated MSM *a*-Ga_2O_3 SBPD is much

larger than that of the post-annealed (PA) MSM *a*-Ga_2O_3 SBPD, whereas the photocurrent is lower. According to the time-dependent photoresponse characteristics, the as-fabricated MSM *a*-Ga_2O_3 SBPD exhibits longer recovery time with τ_{d1}/τ_{d2}=22/383 ms. Based on the noise spectrum of the device, the specific detectivity (D^*) was calculated to be 3.9×10^{12} Jones at 5 V bias voltage at 1kHz. As is presented in Figure S6f, the as-fabricated MSM *a*-Ga_2O_3 PD also exhibits solar-blind photodetection characteristics, but the rejection ratio is much smaller than that of the PA MSM *a*-Ga_2O_3 SBPD. Hence, the PA MSM *a*-Ga_2O_3 SBPD shows much higher performance than the as-fabricated MSM *a*-Ga_2O_3 SBPD.

Table S1. List of key figures-of-merit of the as-fabricated MSM *a*-Ga_2O_3 SBPD at V=5 V under 254 nm light illumination with light intensity of 70 μW/cm^2.

Parameter	As-fabricated MSM *a*-Ga_2O_3 SBPD
I_{dark} (pA)	5.43
I_{photo} (μA)	0.19
R (A/W)	14
D^* (Jones)	3.9×10^{12}
PDCR	3.5×10^4
EQE (%)	6.9×10^3%
R_{250nm}/R_{400nm}	37
$\tau_r/\tau_{d1,}\ \tau_{d2}$ (ms)	5/22,383

Table S2. Comparison between previously reported Ga_2O_3 PDs and our PA MSM *a*-Ga_2O_3 SBPDs.

Photodetectors	I_{dark} (pA)	*R* (A/W)	D^* (Jones)	$R_{uv}/R_{visible}$	*PDCR*	τ_d (ms)	Reference
MSM-*a*-Ga_2O_3	170	8.9	3.3×10^{13}	10^3	–	0.3/1.7	[1]

MSM-β-Ga_2O_3	–	54.9	3.71×10^{14}	3.22×10^{3}	5.58×10^{4}	4000	[2]
MSM-β-Ga_2O_3	14	150	–	–	10^{5}	–	[3]
β-Ga_2O_3/NiO	2.82	0.415	2.27×10^{11}	–	–	–	[4]
MSM-Ga_2O_3: Zn	16	1.05	4.9×10^{11}	–	3.31×10^{5}	2200	[5]
MSM-*a*-Ga_2O_3	200	45.11	–	–	–	0.148	[6]
MSM-*a*-Ga_2O_3	338.6	70.26	1.26×10^{14}	10^{5}	–	20/350	[7]
MSM-β-Ga_2O_3	1.43	96.13	–	–	6.13	32/78	[8]
GaN/Sn: Ga_2O_3	18	3.05	1.69×10^{13}	5.9×10^{3}	10^{4}	18	[9]
β-Ga_2O_3/NSTO	4×10^{4}	43.31	–	–	20	70	[10]
n-Ga_2O_3/p-CuSCN	1.03	0.013	9.43×10^{11}	1.15×10^{4}	4.14×10^{4}	35	[11]
a-/crystalline Ga_2O_3	0.1	0.81	5.67×10^{14}	–	10^{7}	0.012	[12]
p-NiO/n-Ga_2O_3	170	5.7×10^{-5}	5.45×10^{9}	–	122	3650	[13]
a-Ga_2O_3 phototransistor	100	5.67×10^{3}	1.87×10^{15}	–	5×10^{7}	–	[14]
β-Ga_2O_3 phototransistor	0.12	2.6×10^{3}	9.7×10^{13}	–	6×10^{6}	9700	[15]
β-Ga_2O_3 photodiode	–	9.78	3.29×10^{14}	10^{4}	–	5×10^{-3}	[16]
MSM-p-Ga_2O_3	200	9.5×10^{3}	1.5×10^{15}	6×10^{3}	10^{5}	10/40	[17]
MSM-*a*-Ga_2O_3	0.3	733	3.9×10^{16}	5×10^{3}	3.9×10^{7}	18	This work

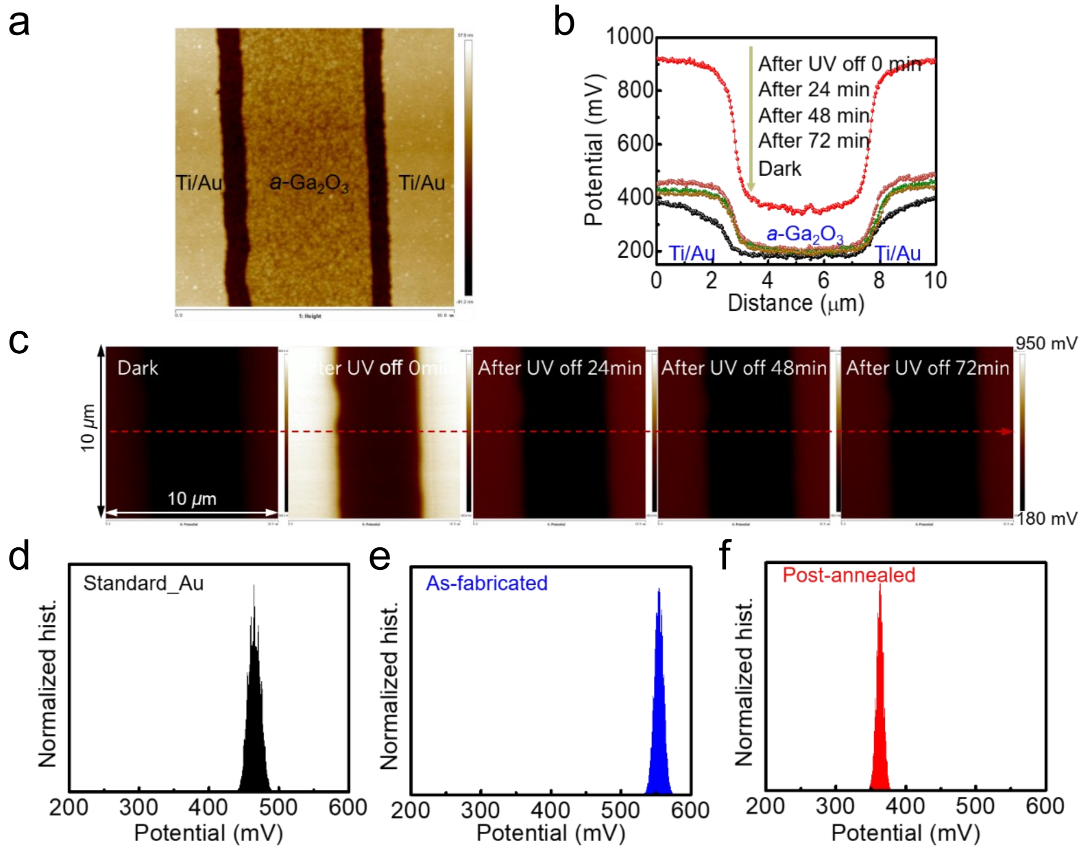

Figure S7. a) AFM image of the scanning area of the MSM *a*-Ga_2O_3 SBPD. b) Surface potential variation process along the red arrow line in Figure S7c. c) Surface potential variation process in a scanning area of 10 μm×10 μm of the as-fabricated *a*-Ga_2O_3 SBPD before and after 254 nm light illumination. Surface potential profile of the d) Au standard sample, e) as-fabricated *a*-Ga_2O_3 film, and f) PA *a*-Ga_2O_3 film.

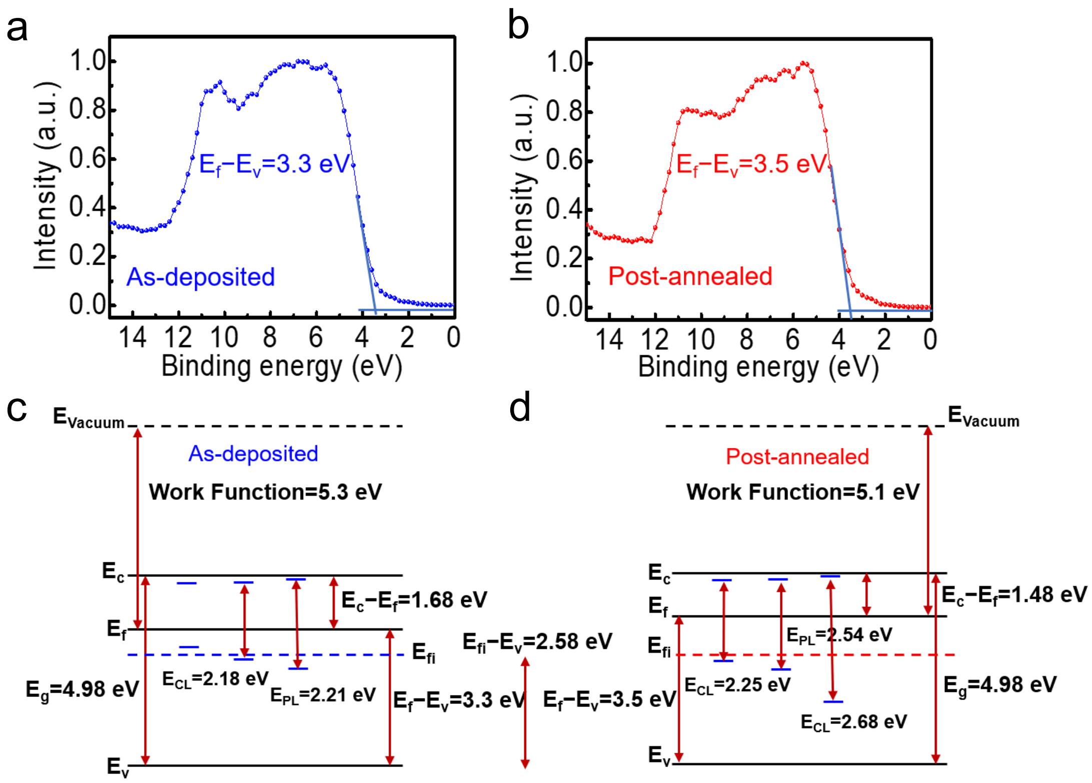

Figure S8. Valance band spectrum for the a) as-deposited and b) PA *a*-Ga_2O_3 film. The energy band diagram of c) as-deposited and d) PA *a*-Ga_2O_3 film.

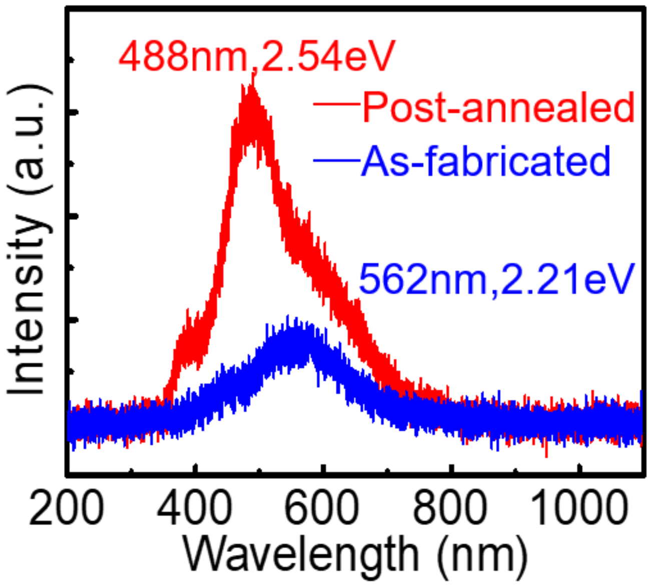

Figure S9. Photoluminescence spectrum of the as-deposited and PA *a*-Ga_2O_3 film.

As shown in Figure S9, broad blue emission peaks were both observed in the photoluminescence (PL) spectrum. The blue emission is considered to occur in Ga_2O_3 by a recombination of electrons at the donor to the hole at the acceptor via neutral defects, generally, the oxygen vacancy. The PL emission intensity of PA MSM *a*-Ga_2O_3 SBPD is much higher than that of the as-fabricated MSM *a*-Ga_2O_3 SBPD, suggesting the enhanced recombination luminescence in the PA *a*-Ga_2O_3 film. This is because that there are more oxygen vacancies in the PA *a*-Ga_2O_3 film, which act as effective recombination center during annihilation process of nonequilibrium carriers. This is one of the reasons that the PA MSM *a*-Ga_2O_3 SBPD exhibits faster recovery speed. The theoretical explanation is further discussed in Supplementary Note 4.

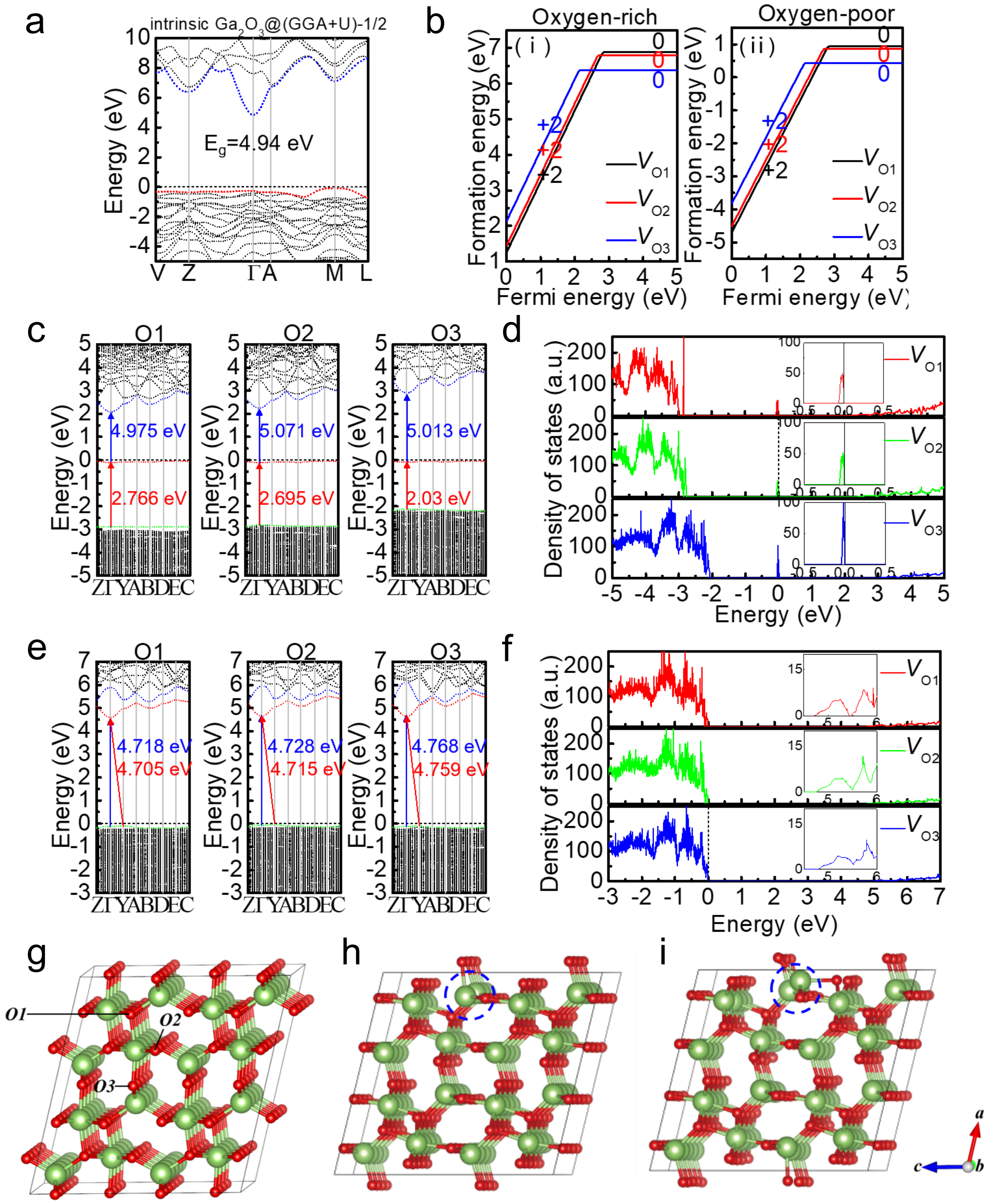

Figure S10. a) The band structure of intrinsic Ga_2O_3 calculated using a (GGA+U)−1/2 method. b) Formation energies for V_O and V_O^{2+} with respect to the Fermi energy under the conditions of i) O-rich and ii) O-poor. Energy band structure diagrams for three kinds of inequivalent oxygen with c) V_O and e) V_O^{2+}. The blue and green dashed lines represent CBM and VBM. The red dashed line is the defect energy level introduced by

oxygen vacancies. The density of states for three inequivalent oxygen atoms in Ga_2O_3 with d) V_O and f) V_O^{2+}. g) Supercell structure of the intrinsic Ga_2O_3. Here O1 is fourfold coordinated, while O2 and O3 are threefold coordinated. Supercell structural deformations of Ga_2O_3 after h) V_O and i) V_O^{2+} relaxation are also given.

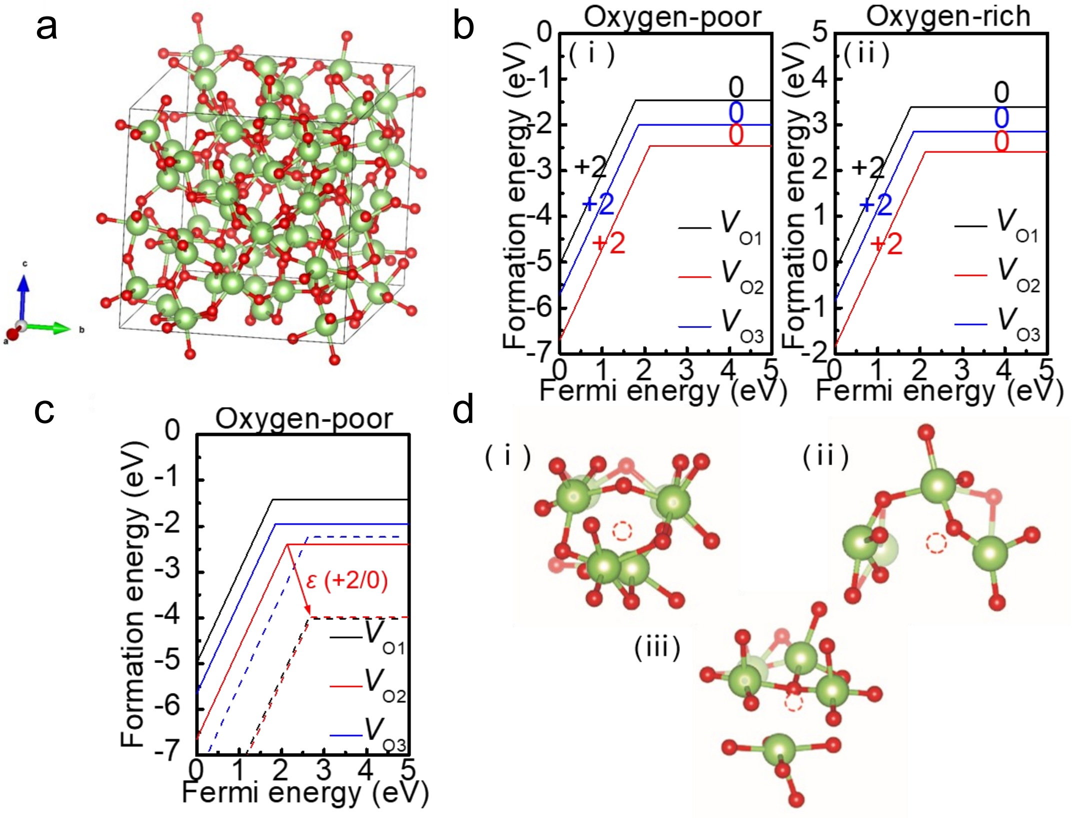

Figure S11. a) Supercell structure of our *a*-Ga_2O_3 model. b) Formation energies of V_O and V_O^{2+} in *a*-Ga_2O_3 with respect to the Fermi energy under the conditions of i) O-poor and ii) O-rich in *a*-Ga_2O_3. c) The variation of formation energy in the amorphous phase before and after annealing under the O-poor condition. The solid and dashed lines represent the formation energies before and after annealing, respectively. d) Atomic structure change around Vo after the annealing process for i) O1, ii) O2 and iii) O3, respectively.

Table S3. The distance variations between the oxygen vacancy position and the neighboring Ga atoms before and after annealing in amorphous Ga_2O_3. (+: move away from oxygen vacancy; −: move toward oxygen vacancy; unit: Å)

	Conditions	Ga1	Ga2	Ga3	Ga4
V_{O1}	Before annealing	0.15	0.11	0.14	0.22
	After annealing	0.37	0.67	−0.18	0.34
V_{O2}	Before annealing	0.17	0.69	0.09	/
	After annealing	0.17	0.68	0.10	/
V_{O3}	Before annealing	0.09	0.02	−0.19	/
	After annealing	0.43	0.14	−0.46	/

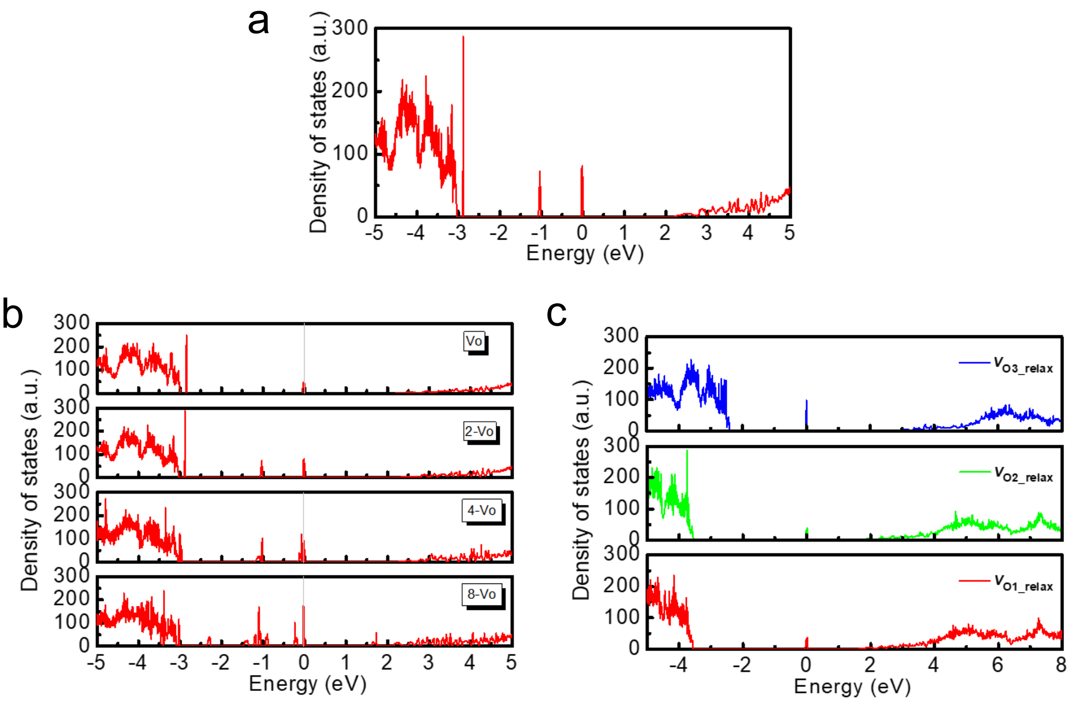

Figure S12. a) Density of states for the Ga_2O_3 supercell with two oxygen vacancies

introduced at the O3 site. b) Density of states for the Ga_2O_3 supercell under various oxygen vacancy concentrations. c) Density of states for the Ga_2O_3 supercell with V_O^{1+} introduced at three kinds of inequivalent oxygen sites, where the structures were relaxed.

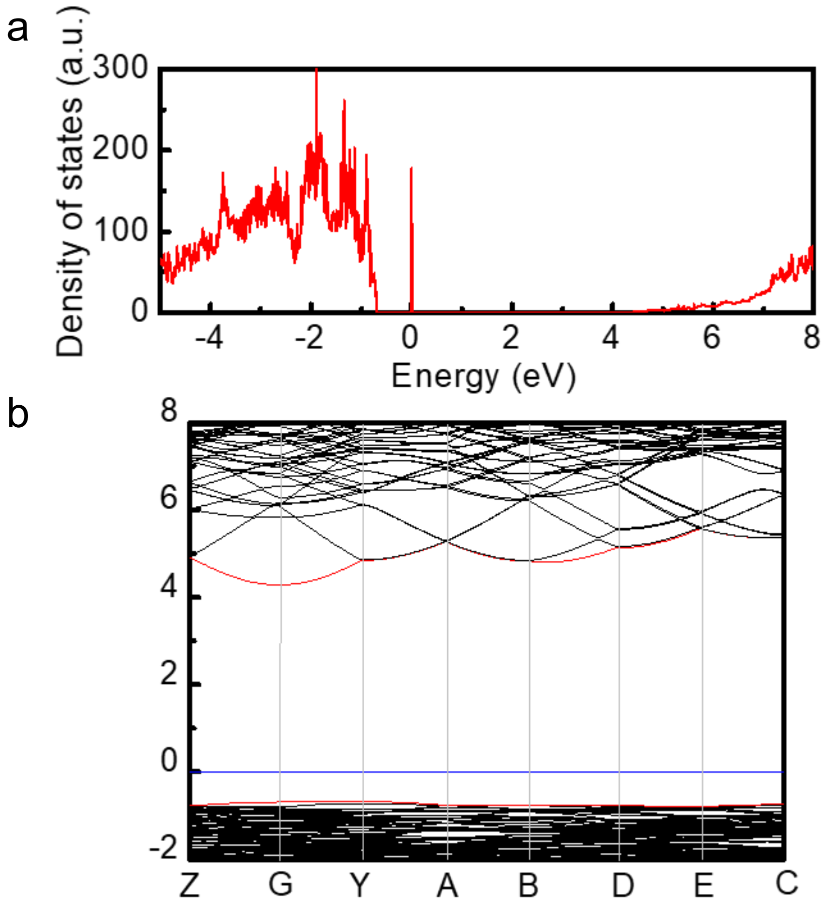

Figure S13. a) Calculated density of states for N-doped Ga_2O_3 and its corresponding energy band structure diagram.

Since the Ga_2O_3 film was annealed in N_2 atmosphere, it is necessary to investigate the status of N in the Ga_2O_3 film. According to our DFT calculation, a deep defect level above the VBM was introduced when N was doped in Ga_2O_3. As shown in Figure S13b,

the defect level is very flat, indicating the effective mass is very large, and the mobility for the trapped electron is very low. This is one of the reasons that the PA MSM *a*-Ga_2O_3 SBPD has lower dark current.

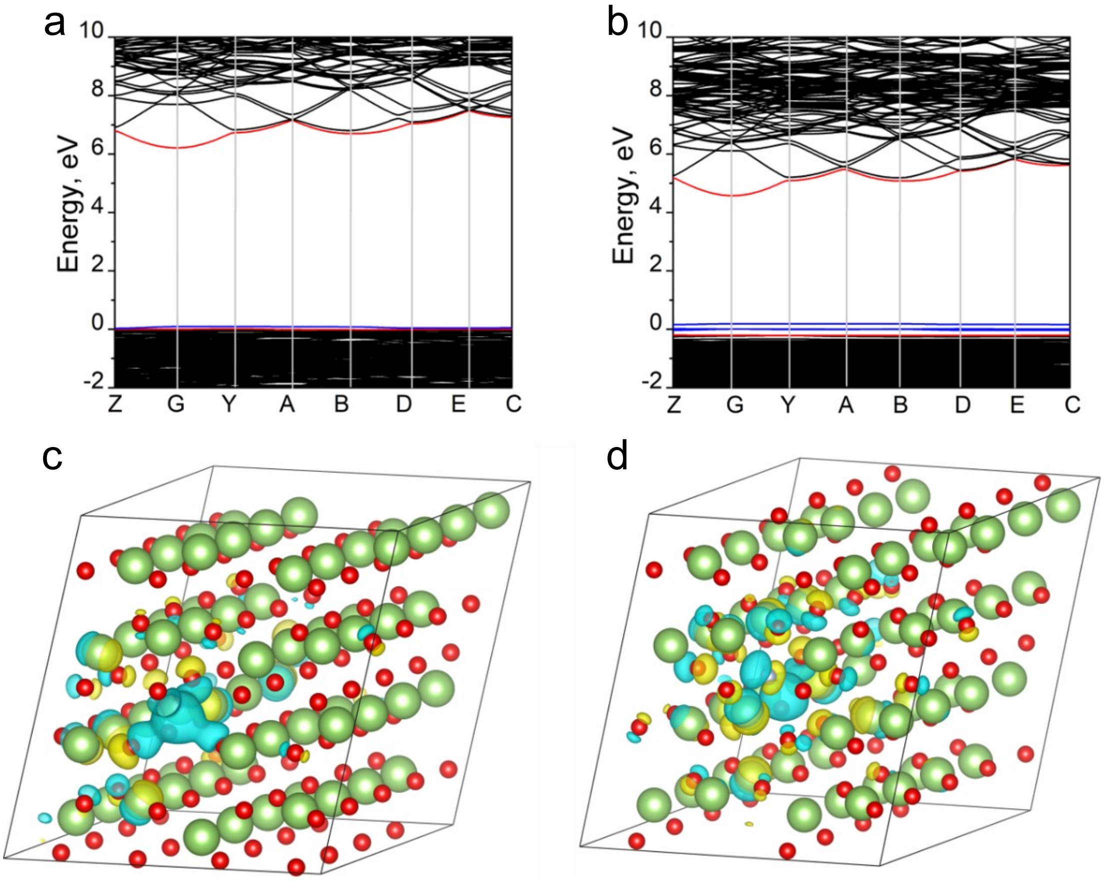

Figure S14.Energy band structure diagrams of a) Ga_2O_3 and b) N-doped Ga_2O_3 with V_{Ga}. Differential charge diagrams of c) Ga_2O_3 and d) N-doped Ga_2O_3 with V_{Ga}. (Yellow bubbles indicate an increase in charge)

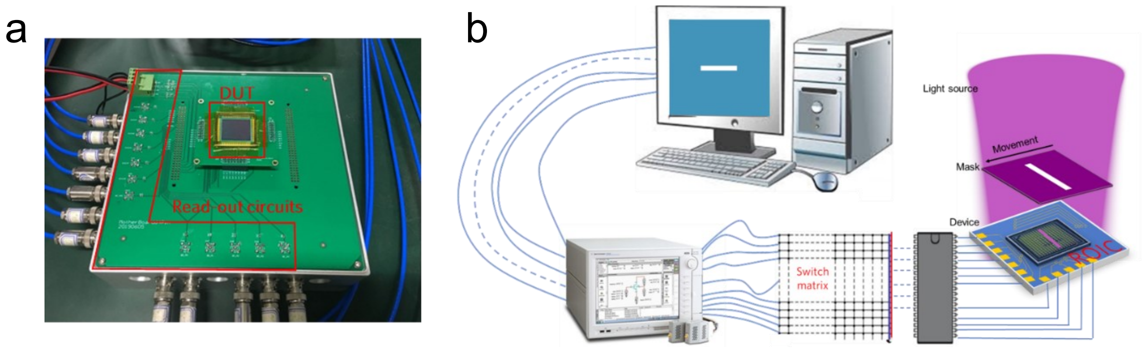

Figure S15. a) Photograph of the packaged image sensor array and read-out circuits. b) Schematic illustration of the homemade 32×32 array imaging system.

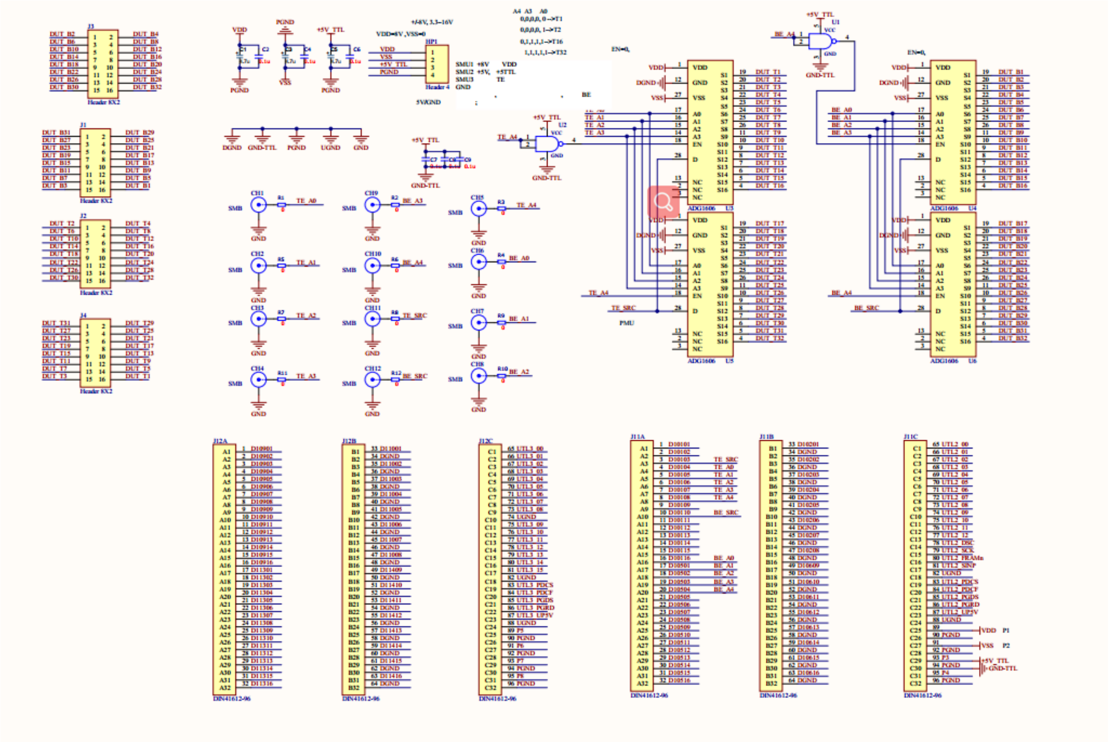

Figure S16. Circuit diagram of the read-out circuits.

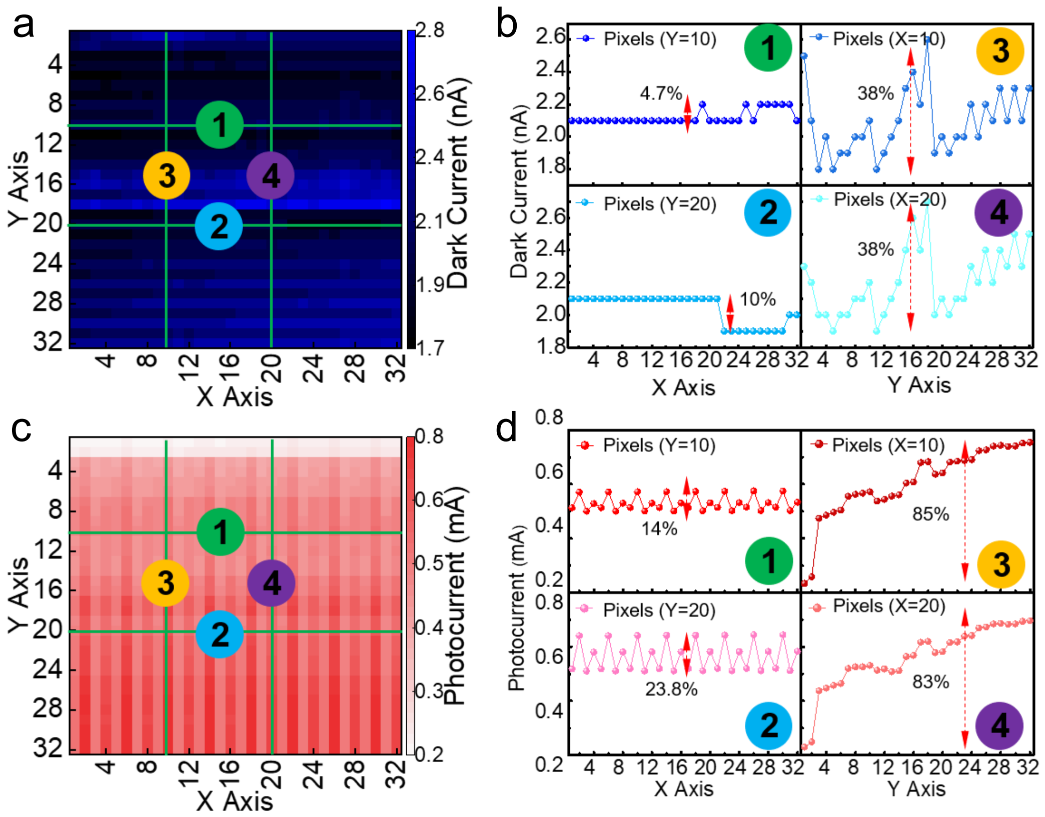

Figure S17. a) Dark current and c) photocurrent mapping of the 32×32 *a*-Ga_2O_3 image sensor array under 254 nm light illumination with 180 μW/cm^2 of light intensity at 4 V. b) Dark current and d) photocurrent profiles measured on four different lines randomly selected in the mapping in a) and c) (1, 2, 3, and 4), respectively.

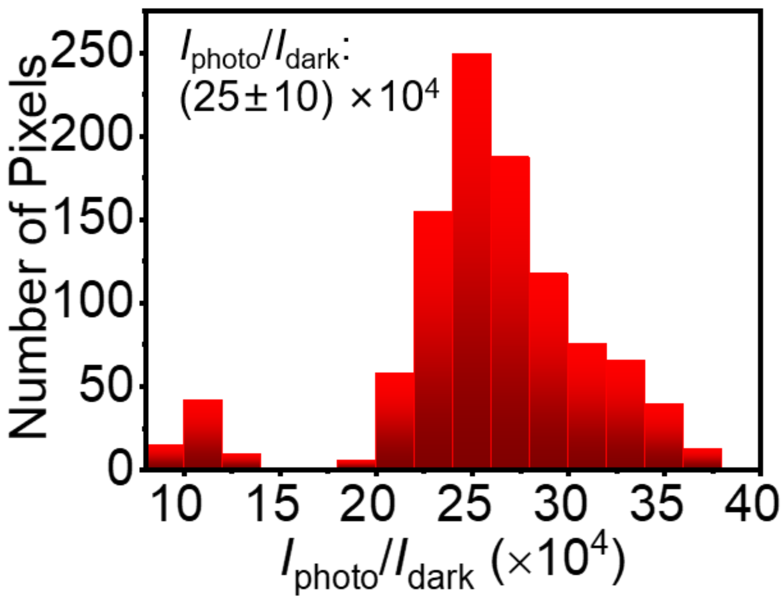

Figure S18. Statistical distribution of I_{photo}/I_{dark} ratio of the array under 4 V bias voltage.

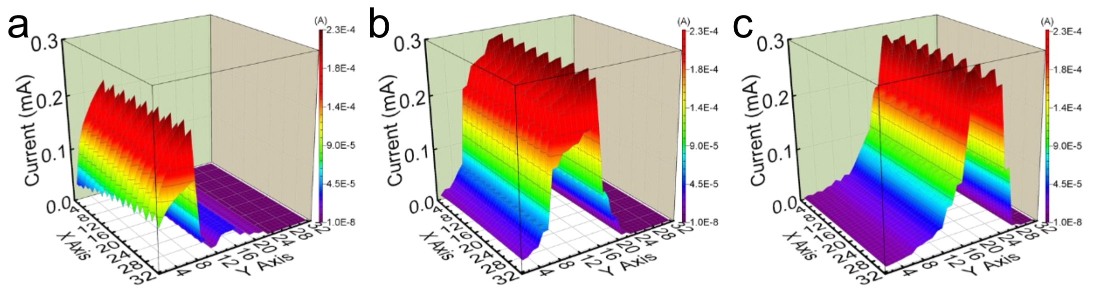

Figure S19.a-c) Three-dimensional photocurrent distribution of all pixels of the light beam in different position in the movement process obtained at 4 V.

Supplementary Note 1. KPFM results of as-fabricated MSM *a*-Ga_2O_3 SBPD and work function calculation

Figure S7a shows the AFM image of the scanning area of the MSM *a*-Ga_2O_3 SBPD. The total scanning area is 10 μm×10 μm with width consisting of 5 μm wide *a*-Ga_2O_3 channel and 2.5 μm wide Ti/Au electrodes on both sides. Figure S7b shows surface

potential variation process along the red arrow line in Figure S7c. It can be found that the CPD of as-fabricated MSM a-Ga_2O_3 SBPD under 254 nm light illumination also increases overall. However, surface CPD in the as-fabricated a-Ga_2O_3 channel region shows less increment compared to that in the PA MSM a-Ga_2O_3 SBPD, which indicates more carriers are photo-generated and trapped in the PA a-Ga_2O_3 film. Besides, surface CPD between Ti/Au metal stacks and as-fabricated a-Ga_2O_3 film became bigger after the device was illuminated with 254 nm light. This may well explain why the photocurrent of as-fabricated MSM a-Ga_2O_3 SBPD is much smaller than that of the PA MSM a-Ga_2O_3 SBPD by the following statements.

As confirmed in the XPS and TEM measurement, the PA a-Ga_2O_3 film has higher concentration of oxygen vacancy in the body area and at the interface of electrodes and a-Ga_2O_3 film. The neutral V_O are tend to be ionized to V_O^{2+} states by the photoexcitation, donating two electrons to the conduction band, which makes contribution to the measured photocurrent.[18, 19] Therefore, more photo-induced carriers will be generated in the PA a-Ga_2O_3 film. Additionally, the Schottky barrier lowering effect is enhanced in the PA a-Ga_2O_3 SBPD. Thus, increased electrons inject to Ga_2O_3 film from metal contact, producing large internal gain in PA MSM a-Ga_2O_3 SBPD.

It is worth noting that the potential distribution curve of the PA MSM a-Ga_2O_3 SBPD is bend up in Figure 4h, whereas the as-fabricated MSM a-Ga_2O_3 SBPD is the opposite. This can be explained by work function difference of the a-Ga_2O_3 film.[20] See details in Figures S7d, e and f. The value obtained from the potential plot is the potential difference V_{CPD} between the probe and the sample. In TP-KPFM-AM mode, the system

defaults the voltage to the tip, so $V_{CPD}=V_{sample}-V_{Tip}$. First, the potential difference V_{CPD} between the probe and Au standard sample was measured. The work function of Au standard sample is 5.2 eV. Therefore, the potential on the tip can be calculated as follows:

$V_{CPDAu}=V_{Sample}-V_{Tip}$, $V_{CPDAu}=0.465$ V, $V_{Sample}=5.2$ V, So $V_{Tip}=4.735$ V.

The potential difference V_{CPD} between the probe and the PA *a*-Ga_2O_3 film $V_{CPD}=0.363$ V, so $V_{PA}= V_{CPD}+V_{Tip}=5.1$ V. Similarly, the potential difference V_{CPD} between the probe and the as-fabricated *a*-Ga_2O_3 film $V_{CPD}=0.554$ V, so $V_{As\text{-}fabricated}=5.3$ V. Hence, the work function of as-fabricated and PA *a*-Ga_2O_3 film is 5.3 eV and 5.1 eV, respectively. Thus, potential distribution curve of the PA MSM *a*-Ga_2O_3 SBPD in Figure 4h is bend up, whereas the as-fabricated MSM *a*-Ga_2O_3 SBPD in Figure S7b is the opposite.

Supplementary Note 2. Energy band information of as-deposited and PA *a*-Ga_2O_3 film

In order to obtain the electronic band structures such as the band gap of the *a*-Ga_2O_3 film and the valance band information, we performed transmittance, X-ray photoelectron spectroscopy valence band, and Kelvin probe measurements. According to transmittance spectrum, the band gap of as-deposited and PA *a*-Ga_2O_3 film are both extracted to be 4.98 eV.

For acquiring the position of Fermi level in the band-gap, XPS measurement was performed. The extrapolation of leading edge to background line provided the valence

band maximum (VBM) position and the binding energy corresponds to E=0 eV represents the Fermi level position relative to the VBM whereas the area under the curve between background line and VBM represents the density of states of acceptors. The energy difference between VBM and Fermi level for as-deposited and PA *a*-Ga_2O_3 film are calculated to be 3.3 eV and 3.5 eV, respectively. The area under the curve between background line and VBM for as-deposited and PA *a*-Ga_2O_3 film are 0.3 and 0.46, respectively, suggesting that there are more acceptor-like defect states near the VBM in the PA *a*-Ga_2O_3 film.

For the work function, KPFM measurements were performed. The work function for as-deposited and PA *a*-Ga_2O_3 film are calculated to be 5.3 eV and 5.1 eV, respectively. Besides, the sub-gap defect states were also obtained by CL and PL measurements. Based on these measurements, the band information of the as-deposited and PA *a*-Ga_2O_3 film are presented in Figures S8 c and d. As was stated in the DFT calculation, V_O^{2+} are more easily to be produced in the PA *a*-Ga_2O_3 film, which may result in lower work function of the PA *a*-Ga_2O_3 film.

Supplementary Note 3. DFT calculations on Ga_2O_3 and the corresponding analysis

First of all, the crystalline *β*-Ga_2O_3 model was used to analyze the formation energies of different oxygen vacancies and their effects on the electronic structure. And the amorphous model was used to simulate the annealing process. In all calculations, the AM05 functional was adopted,[21] which in general yields accurate lattice parameters. For electronic structure calculations, the shGGA−1/2 method based on a GGA+U

ground state was employed,[22, 23] which yields a 4.94 eV bandgap. The calculated bandgap value is in excellent agreement with the experimental value of 4.98 eV. The crystalline supercell structure of Ga_2O_3 used in our DFT calculations is shown in Figure S10g. We have calculated the formation energies of three different kinds of oxygen vacancies under both O-rich and O-poor conditions, using the method as described in the work of Matsunaga et al.[24] Here, O1 is fourfold coordinated, while O2 and O3 are threefold coordinated. The formation energy results (shown in Figure S10b) show that only two kinds of oxygen vacancies can stably exist in Ga_2O_3, namely, neutral oxygen vacancy (V_O) and +2 charged oxygen vacancy (V_O^{2+}). When the Fermi level rises beyond a certain value, the stable charge state changes from V_O^{2+} to V_O. This value is called the charge transfer level (ε (+2/0)). Neutral oxygen vacancies are more stable at higher Fermi levels, and the ε (+2/0) values of V_{O1}, V_{O2}, and V_{O3} are 2.569 eV, 2.316 eV, and 2.094 eV above the VBM, respectively.

Subsequently, we calculated the electronic structure of defective Ga_2O_3 with V_O and V_O^{2+} introduced, respectively. The energy band diagrams and electronic density of states (DOS) of Ga_2O_3 with V_O are presented in Figure S10c and d, respectively. With the presence of V_O on O1, O2 or O3 sites, Ga_2O_3 has direct band gap characteristics (at the Γ point), with band gap values of 4.975 eV, 5.071 eV and 5.013 eV, respectively. The formation of V_O will introduce defect levels near the mid-gap, as shown in Figure S10c (red dashed line). The introduced defect levels are located at 2.766 eV, 2.695 eV and 2.030 eV above the VBM for O1, O2 and O3, respectively. These values are consistent with the charge transfer levels (ε (+2/0)) for all inequivalent oxygen sites

(2.569 eV, 2.316 eV and 2.094 eV, respectively). Therefore, ε (+2/0) reflects the gap between the VBM and the defect levels introduced by the oxygen vacancies. And the closer ε (+2/0) is to CBM, the easier it is for the formation of V_O^{2+}. In these defect states, electrons are found to be strongly localized with strong and narrow DOS as illustrated in Figure S10d.[19, 25] Photoexcited holes are likely to be captured by these acceptor-like traps. In the region of metal electrodes, this will cause the lowering of barrier height, and thus contributing to the high internal gain of the PA MSM a-Ga_2O_3 SBPD.[26-28] Similarly, the energy band diagrams and the DOS of the Ga_2O_3 film with ionized V_O^{2+} are shown in Figure S10e and f, respectively. When the V_O is ionized to V_O^{2+}, the introduced defect level moves toward the CBM, lying at 4.705 eV, 4. 715 eV and 4.759 eV above the VBM for O1, O2 and O3, respectively. In the inset of Figure S10f, relatively high DOS is discovered near the CBM. This difference between V_O and V_O^{2+} can be explained by structural deformation in the supercell of Ga_2O_3 after oxygen vacancy relaxation, as presented in Figures S10h and i. Under the influence of V_O, the surrounding Ga atoms will move close to the oxygen vacancy site, leading to stronger interactions between the Ga atoms. The defect states mainly derive from these interactions. The defect level is found to be near the mid-gap. For the case of V_O^{2+}, however, the surrounding Ga atoms will move outward. And the strength of Ga−O bond is enhanced so that the formation energy is reduced, raising the unoccupied defect level toward the CBM.

To better recover the realistic experimental situation, we also calculated the formation energy of oxygen vacancies in amorphous Ga_2O_3. To generate the a-Ga_2O_3

in this work, DFT-based *ab initio* molecular dynamics (AIMD) simulations were performed.[29, 30] The temperature and time step were respectively set to 3000 K and 5 ns during AIMD simulations, and structure relaxation was carried out to reduce the internal stress. On that basis, the formation energies of oxygen vacancies in *a*-Ga_2O_3 were calculated and the PA of *a*-Ga_2O_3 with oxygen vacancies was simulated. The temperature and time step were respectively set to 675 K and 5 ns for the PA simulation. The calculation results are similar to the results obtained in single crystal β-Ga_2O_3. The supercell structure of amorphous Ga_2O_3 is shown in Figure S11a. Formation energies of different kinds of oxygen vacancies for three inequivalent sites under both O-rich and O-poor conditions were calculated. O1 is fourfold coordinated, while O2 and O3 are threefold coordinated. Similar to single crystalline Ga_2O_3, only neutral oxygen vacancy (V_O) and +2 charged oxygen vacancy (V_O^{2+}) may be stable in Ga_2O_3, as shown in Figure S11b. The effect of annealing at high temperature was further simulated in the DFT calculations. Taking oxygen-poor condition as an example, the formation energies of V_O and V_O^{2+} at various Fermi energy levels before and after annealing are plotted in in Figure S11c. The formation energies of V_O and V_O^{2+} are both reduced, suggesting the oxygen vacancies are more stable after annealing. However, the formation energy of V_O^{2+} is reduced more substantially and ε (+2/0) of $V_{O1}/V_{O2}/V_{O3}$ move toward the CBM (from 1.78/2.12/1.85 eV to 2.63/2.70/2.62 eV, respectively), indicating that V_O^{2+} is created more easily after annealing. It is known that V_O can be ionized to V_O^{2+} under photoexcitation, which acts as shallow donor level resonant with the conduction band.[19, 31, 32] The oxygen vacancies also play a vital role in accelerating

the electron-hole recombination, which will be discussed in detail in Supplementary Note 4. Therefore, the PA MSM *a*-Ga_2O_3 SBPD can achieve higher photocurrent and faster recovery speed.

Another effect of annealing for Ga_2O_3 is structural reconstruction and diffusion. As shown in Figure S11d, the atomic structure relaxation proceeds after the formation of V_O on O1, O2 or O3 sites. Red dashed circles denote the oxygen vacancies, and the virtualized and materialized atoms indicate the atomic locations before and after the annealing process, respectively. We observe that the surrounding oxygen atoms tend to move to the vacancy sites, annihilating the original vacancy sites. On the other hand, Ga atoms tend to move away from oxygen vacancies, which is enhanced after annealing. As listed in Table S2, the distance variations between the oxygen vacancies and neighboring Ga atoms are calculated. For O1, the displacement of coordinated Ga2 atom away from V_O after annealing is 0.67 Å, much larger than that before annealing (0.11 Å). According to previous analysis, such outward displacement strengthens the Ga-O bond, raising the defect level to CBM. This is the reason why V_O^{2+} is more energetically favorable after annealing.

Supplementary Note 4. Discussion of recombination model

From the XPS and TEM measurement, we can find that the post annealing process would increase the concentration of oxygen vacancies in Ga_2O_3. It is helpful to figure out which kind of oxygen vacancy tends to emerge in this process, and the distribution of defect levels. In DFT calculation, we first calculated the DOS of Ga_2O_3 with two

oxygen vacancies. As shown in Figure S10b, since the formation energies of oxygen vacancy in O1 and O2 are similar, we just calculated the formation energy of two oxygen vacancies in three combinations of O2−O2, O2−O3, and O3−O3. The formation energies were 9.36 eV, 8.76 eV and 8.14 eV for O2−O2, O2−O3, and O3−O3, respectively. Hence, with the increase of oxygen vacancy concentration, the O3-vacancy is found to be easier to coexist in Ga_2O_3 with the lowest formation energy. Besides, the defect level moves deeper in the energy gap. This trend becomes more obvious when we further increase the number of oxygen vacancies, as exhibited in Figure S12b. More and more deep defect levels form in the energy gap with the increase of vacancy concentration, instead of moving to the CBM. This is one of the reasons why the PA MSM *a*-Ga_2O_3 SBPDs have lower dark current than that of as-fabricated devices. Additionally, holes are easily to be trapped by these deep defect levels, contributing to the internal gain of the PA MSM *a*-Ga_2O_3 SBPD.

As stated above, the PA MSM *a*-Ga_2O_3 SBPD shows faster recovery speed. This is because that there are more oxygen vacancies in the PA *a*-Ga_2O_3 film, specifically the neutral vacancies, which act as effective recombination centers during the annihilation process of nonequilibrium carriers. In the transient photoresponse characteristic curves, the devices exhibit both a fast component and a slow component in the decay process. The fast component is associated with the band-to-band carrier recombination, while the slow component is related to carrier recombination via defect levels. As can be seen in Figure 3c and Figure S6c, the fast components of the decay process in time-dependent characteristics of PA MSM *a*-Ga_2O_3 SBPD and as-fabricated MSM *a*-Ga_2O_3

SBPD show little difference. Hence, the V_O-related recombination center plays a key role in the decay process of PDs.

Here we shall discuss the V_O-assisted carrier recombination model using first-principles calculation. In the CL and PL spectra, the blue emission peaks were attributed to donor-acceptor pairs recombination. Through DFT calculation, the V_O introduced defect levels were found to reside in the mid-gap, where electrons are found to be highly localized. The holes are easily trapped by these localized states under photoexcitation. When the V_O traps a hole, it becomes V_O^+, and the defect level moves to ~1.5 eV below the CBM as shown in Figure S12c. Since V_O^+ is unstable, it might capture holes or electrons to yield V_O^{2+} or V_O, respectively. However, the defect level is near the CBM, thus it tends to capture electrons from CBM. It should be noted that the V_O^+ will not donate electrons to the CBM, because it is a deep donor level and can only obtain electrons from V_O^{2+} to return to V_O when the light illumination is off. Once electrons are captured by V_O^+, recombination of electrons at CBM and holes at VBM occurs. According to the energy band structures for Ga_2O_3 with V_O or V_O^{2+} introduced in three kinds of inequivalent oxygen sites (see Figures S10c and S10e), the energy level difference between V_O^{2+} and V_O is consistent with the energies of emission peaks in the CL and PL spectra. With the formation of deeper V_O defect levels after annealing, the energy level difference between V_O^{2+} and V_O becomes larger. Correspondingly, emission peaks of CL and PL spectra are of higher intensity and show blue shift in the annealed Ga_2O_3 film.

Supplementary Note 5. Discussion of Ga vacancy

By adjusting the U value in the DFT calculation, we calculated the defect levels introduced by V_{Ga}. As presented in Figures S14 a and S14b, the defect levels are close to VBM, about 0.18 eV and 0.41 eV above VBM for pure Ga_2O_3 and N-doped Ga_2O_3, respectively. According to the differential charge diagrams (Figure S14 c and d), after the formation of V_{Ga} the surrounding oxygen atoms will get more electrons from other Ga atoms, and the bonding will be strengthened. The electron is localized near the oxygen atom (in the opposite direction of the V_{Ga}). After V_{Ga} is formed near N atoms, the surrounding oxygen atoms still get more electrons from other Ga atoms, while electrons around nitrogen atoms gather toward V_{Ga}, forming defect levels. These defect states are not good recombination centers and do not match the CL and PL results.

Supplementary Note 6. Computational details of DFT calculation

The DFT calculations were carried out using the plane-wave-based Vienna *Ab initio* Simulation Package (VASP 5.4.4).[33, 34] All calculations were based on a 1×4×2 *β*-Ga_2O_3 supercell that includes 64 Ga atoms and 96 O atoms. The generalized gradient approximation (GGA) functional with the Armiento-Mattsson 2005 (AM05) flavor was employed to account for the exchange-correlation energy,[21] since it is known to predict accurate lattice constants. The valence electron configurations were 4s and 4p for Ga; 2s and 2p for O. Projector augmented-wave pseudopotentials were used to replace the

core electrons. The plane wave kinetic energy cutoff was fixed to be 500 eV. For all structural relaxations, the convergence criterion in electronic self-consistent runs was set to 10^{-6} eV, and structural optimization was obtained until the Hellmann-Feynman force acting on any atom was less than 0.02 eV/Å in each direction. The Brillouin zones were sampled by a 3×3×3 equal-spacing k-point mesh. The electronic structure calculation for β-Ga_2O_3 is quite challenging in that it involves closed shell 3d electrons that cannot be described well by DFT-GGA. Hence, a standard 8 eV on-site Hubbard correction was applied to the 3d orbitals of Ga. Subsequently, we employed an efficient self-energy correction scheme (DFT−1/2) originally proposed by Ferreira et al., and later improved by Xue et al. (shDFT−1/2).[22, 23] The *ab initio* molecular dynamics (AIMD) technique was utilized to establish the amorphous Ga_2O_3 supercell, which was used to analyze the defect formation energy in amorphous Ga_2O_3. For the simulation of the annealing process, the annealing temperature was set to 675 K, and heat-up time was set to 5 ns.

Supplementary Note 7. Evaluation of uniformity test of the image sensor array

As shown in Figures S16 a and c, dark and photocurrent mapping of the image sensor array are obtained at V= 4 V in the dark and under 180 μW/cm^2 254 nm light illumination, respectively. In order to better characterize the uniformity, current distribution along randomly chosen four lines in Figures S15a and c are extracted and plotted in Figures S16b and d. The dark current is observed to show a minimum

fluctuation of 4.7% along line 1, defined as the difference between the maximum and minimum values divided by the average value of the current. The maximum fluctuation of dark current is about 38%, suggesting relatively satisfactory dark current uniformity. Under 180 μW/cm^2 254 nm light illumination, photocurrent of hundreds of μA was measured in all pixels, demonstrating a high current contrast of ~10^4. As presented in Figure S16d, the photocurrent shows large fluctuation, as high as 20% along line 3 and 4 in Figure S16c, which indicates the uniformity of photocurrent response of the image sensor array should be further improved in the future. Figure S17 demonstrates statistical distribution of I_{photo}/I_{dark} ratio of the array under 4 V bias voltage. The statistical of I_{photo}/I_{dark} ratio is about $(25\pm10)\times10^4$. Overall, the fabricated image sensor array based on post-annealed MSM a-Ga_2O_3 SBPDs demonstrates satisfactory sensor uniformity for image generation and recognition. It should be noted that the dark current of all pixels in the array are relatively larger than that of single device, and the current of some neighboring unilluminated pixels in Figure 5d are larger than the dark current presented in Figure S16a. Additionally, the output images in Figure 5d show unsharp shape of the light beam. This is probably because of cross-talk issue due to existence of current sneak path in the array. One possible way to solve this problem is to adopt one transistor-one photodetector (1T−1PD) or one diode-one photodetector (1D−1PD) structure.[35, 36]

References

[1] Y. Chen, Y. Lu, M. Liao, Y. Tian, Q. Liu, C. Gao, X. Yang, C. Shan, *Adv. Funct. Mater.* **2019**, *0*, 1906040.

[2] L.-X. Qian, H.-F. Zhang, P. T. Lai, Z.-H. Wu, X.-Z. Liu, *Opt. Mater. Express* **2017**, *7*, 3643.

[3] Y. Xu, Z. An, L. Zhang, Q. Feng, J. Zhang, C. Zhang, Y. Hao, *Opt. Mater. Express* **2018**, *8*, 2941.

[4] K.-H. Li, N. Alfaraj, C. H. Kang, L. Braic, M. N. Hedhili, Z. Guo, T. K. Ng, B. S. Ooi, *ACS Appl. Mater. Interfaces* **2019**, *11*, 35095.

[5] Y. Li, D. Zhang, R. Lin, Z. Zhang, W. Zheng, F. Huang, *ACS Appl. Mater. Interfaces* **2019**, *11*, 1013.

[6] S. H. Lee, S. B. Kim, Y.-J. Moon, S. M. Kim, H. J. Jung, M. S. Seo, K. M. Lee, S.-K. Kim, S. W. Lee, *ACS Photonics* **2017**, *4*, 2937.

[7] L.-X. Qian, Z.-H. Wu, Y.-Y. Zhang, P. T. Lai, X.-Z. Liu, Y.-R. Li, *ACS Photonics* **2017**, *4*, 2203.

[8] K. Arora, N. Goel, M. Kumar, M. Kumar, *ACS Photonics* **2018**, *5*, 2391.

[9] D. Guo, Y. Su, H. Shi, P. Li, N. Zhao, J. Ye, S. Wang, A. Liu, Z. Chen, C. Li, W. Tang, *ACS Nano* **2018**, *12*, 12827.

[10] D. Guo, H. Liu, P. Li, Z. Wu, S. Wang, C. Cui, C. Li, W. Tang, *ACS Appl. Mater. Interfaces* **2017**, *9*, 1619.

[11] S. Li, D. Guo, P. Li, X. Wang, Y. Wang, Z. Yan, Z. Liu, Y. Zhi, Y. Huang, Z. Wu, W. Tang, *ACS Appl. Mater. Interfaces* **2019**, *11*, 35105.

[12] Y. Wang, W. Cui, J. Yu, Y. Zhi, H. Li, Z.-Y. Hu, X. Sang, E.-j. Guo, W. Tang, Z. Wu, *ACS Appl. Mater. Interfaces* **2019**, *11*, 45922.

[13] Y. Wang, C. Wu, D. Guo, P. Li, S. Wang, A. Liu, C. Li, F. Wu, W. Tang, *ACS Appl. Electron. Mater.* **2020**.

[14] Z. Han, H. Liang, W. Huo, X. Zhu, X. Du, Z. Mei, *Adv. Opt. Mater.* **2020**, *8*, 1901833.

[15] S. Kim, S. Oh, J. Kim, *ACS Photonics* **2019**, *6*, 1026.

[16] Y. Xu, X. Chen, Y. Zhang, F. Ren, S. Gu, J. Ye, *IEEE Electron Device Lett.* **2020**, *41*, 997.

[17] Z. X. Jiang, Z. Y. Wu, C. C. Ma, J. N. Deng, H. Zhang, Y. Xu, J. D. Ye, Z. L. Fang, G. Q. Zhang, J. Y. Kang, T. Y. Zhang, *Mater. Today Phys.* **2020**, *14*, 100226.

[18] B. Ryu, H.-K. Noh, E.-A. Choi, K. J. Chang, *Appl. Phys. Lett.* **2010**, *97*, 022108.

[19] S. Jeon, I. Song, S. Lee, B. Ryu, S.-E. Ahn, E. Lee, Y. Kim, A. Nathan, J. Robertson, U. I. Chung, *Adv. Mater.* **2014**, *26*, 7102.

[20] W. Melitz, J. Shen, A. C. Kummel, S. Lee, *Surf. Sci. Rep.* **2011**, *66*, 1.

[21] R. Armiento, A. E. Mattsson, *Phys. Rev. B* **2005**, *72*, 085108.

[22] K.-H. Xue, J.-H. Yuan, L. R. C. Fonseca, X.-S. Miao, *Comput. Mater. Sci.* **2018**, *153*, 493.

[23] L. G. Ferreira, M. Marques, L. K. Teles, *Phys. Rev. B* **2008**, *78*, 125116.

[24] K. Matsunaga, T. Tanaka, T. Yamamoto, Y. Ikuhara, *Phys. Rev. B* **2003**, *68*, 085110.

[25] H. Liang, S. Cui, R. Su, P. Guan, Y. He, L. Yang, L. Chen, Y. Zhang, Z. Mei, X. Du, *ACS Photonics* **2019**, *6*, 351.

[26] O. Katz, V. Garber, B. Meyler, G. Bahir, J. Salzman, *Appl. Phys. Lett.* **2001**, *79*, 1417.

[27] S. Rathkanthiwar, A. Kalra, S. V. Solanke, N. Mohta, R. Muralidharan, S. Raghavan, D. N. Nath, *J. Appl. Phys.* **2017**, *121*, 164502.

[28] J. B. Varley, A. Janotti, C. Franchini, C. G. Van de Walle, *Phys. Rev. B* **2012**, *85*, 081109.

[29] M. E. Tuckerman, *J. Phys.: Condens. Matter* **2002**, *14*, R1297.

[30] T. D. Kühne, M. Krack, F. R. Mohamed, M. Parrinello, *Phys. Rev. Lett.* **2007**, *98*, 066401.

[31] S. Jeon, S.-E. Ahn, I. Song, C. J. Kim, U. I. Chung, E. Lee, I. Yoo, A. Nathan, S. Lee, K. Ghaffarzadeh, J. Robertson, K. Kim, *Nat. Mater.* **2012**, *11*, 301.

[32] S.-E. Ahn, I. Song, S. Jeon, Y. W. Jeon, Y. Kim, C. Kim, B. Ryu, J.-H. Lee, A. Nathan, S. Lee, G. T. Kim, U. I. Chung, *Adv. Mater.* **2012**, *24*, 2631.

[33] G. Kresse, J. Furthmüller, *Phys. Rev. B* **1996**, *54*, 11169.

[34] G. Kresse, J. Furthmüller, *Comput. Mater. Sci.* **1996**, *6*, 15.

[35] T.-W. Kim, H. Choi, S.-H. Oh, G. Wang, D.-Y. Kim, H. Hwang, T. Lee, *Adv. Mater.* **2009**, *21*, 2497.

[36] J. Kim, J. Kim, S. Jo, J. Kang, J.-W. Jo, M. Lee, J. Moon, L. Yang, M.-G. Kim, Y.-H. Kim, S. K. Park, *Adv. Mater.* **2016**, *28*, 3078.
